# A Mixed Methods Approach to Exploring the Relationship between Norway Rat (*Rattus norvegicus*) Abundance and Features of the Urban Environment in an Inner-City Neighborhood of Vancouver, Canada

**DOI:** 10.1371/journal.pone.0097776

**Published:** 2014-05-15

**Authors:** Chelsea G. Himsworth, Kirbee L. Parsons, Alice Y. T. Feng, Thomas Kerr, Claire M. Jardine, David M. Patrick

**Affiliations:** 1 School of Population and Public Health, University of British Columbia, Vancouver, British Columbia, Canada; 2 Animal Health Centre, British Columbia Ministry of Agriculture, Abbotsford, British Columbia, Canada; 3 British Columbia Centre for Excellence in HIV/AIDS, St. Paul's Hospital, Vancouver, British Columbia, Canada; 4 Department of Medicine, University of British Columbia, Vancouver, British Columbia, Canada; 5 Department of Pathobiology, University of Guelph, Guelph, Ontario, Canada; Université Pierre et Marie Curie, France

## Abstract

Urban rats (*Rattus* spp.) are among the most ubiquitous pest species in the world. Previous research has shown that rat abundance is largely determined by features of the environment; however, the specific urban environmental factors that influence rat population density within cities have yet to be clearly identified. Additionally, there are no well described tools or methodologies for conducting an in-depth evaluation of the relationship between urban rat abundance and the environment. In this study, we developed a systematic environmental observation tool using methods borrowed from the field of systematic social observation. This tool, which employed a combination of quantitative and qualitative methodologies, was then used to identify environmental factors associated with the relative abundance of Norway rats (*Rattus norvegicus*) in an inner-city neighborhood of Vancouver, Canada. Using a multivariate zero-inflated negative binomial model, we found that a variety of factors, including specific land use, building condition, and amount of refuse, were related to rat presence and abundance. Qualitative data largely supported and further clarified observed statistical relationships, but also identified conflicting and unique situations not easily captured through quantitative methods. Overall, the tool helped us to better understand the relationship between features of the urban environment and relative rat abundance within our study area and may useful for studying environmental determinants of zoonotic disease prevalence/distribution among urban rat populations in the future.

## Introduction

Norway rats (*Rattus norvegicus*) are among the most widespread rodent species, inhabiting every continent except for Antarctica [1]. The ubiquity of this species is largely attributable to their remarkable ability to quickly and successfully adapt to new environments and resources [1]. Norway rats are best suited to close cohabitation with people, and are considered a true commensal species as few populations are found in a truly sylvatic state [1].

Their propensity to exploit human settlements has caused Norway rats to become a particularly ubiquitous and problematic pest in cities around the world. Within urban settings, Norway rats can damage property, contaminate food, and act as a source of infectious disease for people [1], [2]. For these reasons there is considerable interest in identifying and understanding the factors that promote or deter rat infestations in urban centers.

Previous studies have indicated that the environment is probably the single most important determinant of rat density and distribution across the urban landscape [1], [3]. More specifically, it is clear that rats require suitable harborage and food sources in order to establish an infestation [1], [3]. However, the adaptability of Norway rats means that it is often difficult to identify what specific features of the urban environment combine to create optimal rat habitat.

Researchers have employed a variety of methods in an effort to identify environmental features that promote or deter rat infestations. For example, a study in Buenos Aires, Argentina, compared the relative abundance of Norway rats in several locations demonstrating varying degrees of human modification [4]. They found that Norway rats were more common in shantytowns and parklands compared to industrial-residential neighborhoods and natural preserves [4]. Another study in the same city found that Norway rats were more abundant in shantytowns compared to parklands [5]. In Salzburg, Austria, Norway rats were trapped in a variety of urban locations with distinct environmental features [6]. This study found that Norway rats were most common in habitats with water, vegetation, natural soil, and organic waste, while rats were rarely found in heavily built-up areas and areas frequently utilized by people [6].

The main drawback of the aforementioned approaches is that they are capable of capturing only ‘high-level’ environmental impacts on rat populations (i.e., differences in rat abundance between different ecosystems). Rat population density and distribution, however, may vary considerably even over very short geographic distances within a single urban ecosystem [7], [8], suggesting that minute differences in the micro-environment of a city block could determine its capacity to support rat populations. These micro-environmental data, however, are relatively difficult to collect in a systematic fashion, and is seldom captured by cursory observation or pre-established databases.

A similar problem has been encountered in the social and health sciences, where researchers have attempted to identify links between an individual's health, welfare, behaviour, etc., and the features of the neighborhood in which they reside. In order to overcome this problem, researchers have developed the science of systematic social observation (SSO) [9], [10]. The tools employed by SSO require the researcher to closely observe and quantify multiple different aspects of a city block, resulting in the creation of a comprehensive dataset that captures the complexity of the block environment [9], [10]. These tools have revolutionized our understanding of impact of the urban environment on the people who reside within it.

Given that the urban environment also influences rat populations, it seems possible that a similar tool could be developed and used to predict rat abundance. There are several studies that have used a similar approach to study rats. However the tools and methodologies employed by these studies had several limitations including the following: capturing a limited number of environmental features [11], being specific to rural [12] or developing areas [13], [14], and being difficult to reproduce [13], [14].

The goals of this study were: 1) to develop a systematic environmental observational tool that captures and quantifies features of a developed urban microenvironment that could influence rat populations; 2) to develop an approach to using that tool that includes analysis of both quantitative and qualitative data; and 3) to use that tool/approach to identify environmental factors associated with relative rat abundance within an inner-city neighborhood of Vancouver, Canada.

## Methods

This study was a part of a larger project (the Vancouver Rat Project, www.vancouverratproject.com) the goal of which is to characterize the health risks that rats pose to people through the transmission of infectious diseases. More specifically, the project aims to understand the ecology of zoonoses in rats and people by understanding the dynamic interplay among rats, people, pathogens, and the urban environment.

### Ethics Statement

This study was approved by the University of British Columbia's Animal Care Committee (A11-0087) and adhered to national guidelines set out by the Canadian Council on Animal Care (www.ccac.ca), including those pertaining to animal user training, euthanasia, protocol review, and wildlife (http://www.ccac.ca/en_/standards/guidelines). This study did not involve any endangered or protected species.

### Study Area

The study area was composed of 43 contiguous city blocks (0.82 km^2^) which included the core of Vancouver's Downtown Eastside (49°15″0′N/123°8″0′W). The Downtown Eastside (DTES) is an impoverished inner-city neighborhood characterized by a large number of single-room occupancy hotels, high rates of homelessness, and a large open air illicit drug scene [15]. It was selected as a priority for study within the city because residents and community groups identified rat infestations as a significant but neglected issue in the area. Also included in the study were several blocks from the more affluent, adjacent Gastown district. All trapping took place on public property only and no specific permissions were required.

### Development of an Environmental Observation Tool

The environmental observation tool (Appendix S1) was modeled after the SSO tool developed by Parsons et al. (2010). We utilized scales, items, and constructs from SSO tools [10], [16]–[18] that measured factors with the potential to influence rat populations, as well as from prior studies of rat abundance [11], [13], [14]. We also developed new items based on a review of the literature regarding rat ecology [1] and our field observations. The final tool included 58 items covering 6 major domains: (1) land use characteristics, (2) property condition, (3) green space characteristics, (4) alley surface characteristics, (5) presence of waste, and (6) alley use (i.e., for loitering and transportation). Additionally, there was a free form section at the end of the tool where observers could make notes about the block under evaluation. For this free form section, particular attention was paid to identifying and describing features that were perceived to be associated with rat presence/abundance, as well as unusual or unique features of the block under study.

In order to facilitate the use of the tool in this study, and in the future, each domain and item was accompanied by detailed instructions regarding its purpose and/or use. Additionally, a supplementary document was produced with photographic examples of different items and ratings (see Appendix S2).

### Tool Implementation

Two observers were trained in the use of the tool, and these raters completed their observations together between 8:00 h and 16:00 h, with final ratings for each item being arrived at via consensus. To complete their observations, observers evaluated all aspects of the block including block faces (i.e., the area of the block facing a street), alleyways, and aerial photographs.

### Determining Rat Abundance

This study was part of a larger project characterizing the ecology of zoonotic pathogens in rat populations for which whole rat carcasses were required for testing. For this reason a trapping-removal method (vs. a non-lethal method, such as mark-recapture) was required. It should be noted, however, that systematic trapping-removal is considered to be among the most accurate methods for enumerating rats, and has been used frequently in the past to determine rat abundance in urban centers [7], [19]–[21].

Each city block randomly assigned to a 3-week trapping period over the course of 1 year in order to capture seasonal variations in rat abundance within the study as a whole. Of the 43 blocks included in the study, 9 were trapped in the fall, 10 in the winter, 14 in the spring and 12 in the summer.

Within each block, approximately 20 Tomahawk Rigid Traps for rats (Tomahawk Live Trap, Hazlelhurst, USA) per block were set out along each side of the back alley that bisected the block. Traps were evenly spaced where possible, but had to be placed on outdoor public property in a location where they did not obstruct traffic and could be secured to prevent theft. Traps were pre-baited (filled with bait but fixed open) for one week to acclimatize rats to trapping equipment and bait, followed by two weeks of active trapping. Baits used included peanut butter, bacon fat, flour, and oats. Traps were set at 16:00 h and checked the following morning at 8:00 h.

Trapped rats were anesthetized with isoflurane in a rodent Inhalation Narcosis Chamber (Harvard Apparatus, Holliston, USA) prior to pentobarbital euthanasia via intracardiac injection. Rats were identified to species by external morphology [22].

### Dependent Variable

Of the 673 rats trapped over the course of the study, 665 (98.8%) were Norway rats (*Rattus norvegicus*) and 8 (1.2%) were black rats (*Rattus rattus*). Given the relatively small number of black rats trapped, and the fact that the ecology of black rats has been shown to be significantly different than Norway rats [1], black rats were excluded from the analysis.

The unit of analysis was the city block (n  =  43). The dependent variable was trap success rate in each block, with trap success rate being equal to the number of rats trapped in a block over the entire trapping period divided by the total trap effort over that period. The total trap effort (# traps set x # nights) was adjusted according to the method described by Nelson and Clark [23]. This method involves subtracting half a unit from the total trap effort for each trap spring for any cause (e.g., trapping of a rat, trapping of a non-target species, tripping of a trap). This adjustment was considered particularly important because of the marked variation in rat abundance among blocks, and because of the frequency with which traps were sprung by non-target species and by members of the public, both of which can significantly bias trap success indices if they are not taken into account [23].

Given that each block under study had roughly the same geometric surface area (1.2 ha) and was trapped in the same manner, and given that rat populations are thought to be relatively isolated to the level of a city block with minimal movement of animals among blocks [1], it is appropriate to use the city block as the unit of analysis and the number of rats trapped offset by trap effort as a proxy for relative population density/rat abundance.

To confirm the absence of geographic autocorrelation of rat abundance among the blocks, a georeferenced map of block outlines within the study area was imported into ArcGIS 10.0 (ESRI, Redlands, USA). Global Moran's I was used to test the null hypothesis that trap success (# rat trapped/trap effort) within a block was randomly distributed (i.e., no spatial autocorrelation) using inverse distance for conceptualization of spatial relationships and a Euclidean distance method.

### Predictor Variables

A total of 59 potential predictor variables were included for initial consideration. Each of the 58 items on the environmental observational tool was used to create 1 predictor variable (Table S1 – note that variables were numbered according to the item numbering in the observation tool). The season in which the block was trapped (fall  =  September – November, winter  =  December – February, spring  =  March – May, summer  =  June – August) was also included as a predictor.

Seven variables (variable nos. 1, 2, 5, 13, 25, 31, and 41) were eliminated initially because they captured the same and/or less information as subsequent variables. For example, variable 13 is a nominal-categorical variable that characterizes the predominant housing type in a block, with 8 categories that each correspond to a class of housing. However, variable nos. 14–19 are ordinal variables that capture both the presence of each class of housing in a block, as well as the proportion of each block occupied by each class of housing.

Next, variables representing different aspects of the same concept were consolidated into a single new composite variable representing the weighted average of the underlying component variables. For example, variable nos. 26–30 all represent building condition (the underlying concept of interest), and pertain to the proportion of each block occupied by buildings in ‘extremely poor’, ‘poor’, ‘fair’, ‘good’, and ‘excellent’ condition. These variables were combined into a single composite variable, as were variable nos. 32–36 and 42–44.

Predictor variables where > 95% of observations had the same score (variable nos. 8, 12, 18, and 54) were deemed non-discriminatory and removed. Variable no. 46 (number of rat holes) was also removed because it was deemed to be a sign of rat abundance rather than a cause.

A total of 37 predictor variables were considered for statistical modeling.

### Model Building

The goal of the model-building protocol was to identify the most parsimonious set of predictor variables that best explained the outcome, and all predictor variables were given equal consideration going into the model building procedure (i.e., we had no a priori hypotheses regarding the relative importance of specific predictors). Similarly, we had no a priori hypothesis regarding interactions among predictors, and, given the large number of potential predictors (n  =  37) relative to the number of observations (n  =  43), no interaction terms were included for consideration.

A zero-inflated negative binomial (ZINB) model was used to model the relationship between the predictors and outcome in order to account for excess zeros (no rats were trapped in 11 of 43 blocks) and overdispersion in the outcome variable. This ZINB model combined a binary model with a logit link to model the odds of not trapping any rats (zero-inflation model), and a negative binomial model with a log link to model the number of rats trapped offset by the natural logarithm of the trap effort (count model). The unit of analysis was the city block (n  =  43).

The relationship between each predictor and the outcome was first modeled in a bivariate manner, including the predictor in the binary and count portions of the model independently (Table S2). The majority of the predictor variables captured the proportion of the block occupied by an environmental factor of interest, and were therefore modeled in a linear manner. For several ordinal variables (variable nos. 48, 49, 57, and 58) where the spacing between categories was more subjective (e.g., ‘none’ vs. ‘some’ vs. ‘a little’ vs. ‘a lot’), bivariate models treating the variable in a linear and categorical manner were generated and compared using the Bayesian Information Criterion (BIC) [24]. For all 4 predictors, the linear form of the variable was superior to the categorical form in both the binary and count portions of the model, therefore these variables were treated as numeric variables. Season was treated as a categorical variable.

In order to narrow down the pool of potential predictors for inclusion in a multivariate model, a program was created in R (R Development Core Team, Vienna, Austria) that performed automated combined forward and backward selection on each part of the model independently using Akaike's Information Criterion (AIC) to select the best potential group of predictors for further consideration [24]. Briefly, in order to select the binary model, the negative binomial model was set at 1 (offset by the natural logarithm of the trap effort). The null binary model (a binary model with no predictors) was used as the starting point, and predictors were added sequentially based on their associated AIC (i.e., the predictor that produced the lowest AIC for the binary model was added first). After each addition, the model was checked to determine if removal of one or more predictors included in the model affected the AIC. If removal of the predictor(s) decreased the AIC for the binary model, then the predictor(s) was eliminated. If removal increased the AIC, then the predictor(s) was retained. This process was repeated until addition or elimination of any subsequent predictors could not further decrease the binary model AIC. The selection procedure described above was then repeated for the count model holding the binary model at 1. All 37 predictor variables, including season, were allowed to compete equally for both components of the model, with the stepwise selection procedure ensuring that only those variables that improved model fit (according to the AIC) were retained. This process should control for confounding among predictors because it will select the variables with the strongest relationship to the outcome in the presence of all other predictor variables.

Variables identified through automated selection for the binary and count components of the ZINB model were then combined in a single model and manual forward and backward selection was performed using the BIC to maximize model fit and parsimony (as the BIC penalizes model complexity more heavily than the AIC) [24]. The final model was that with the lowest BIC.

To investigate collinearity among explanatory variables in the final model, Spearman's rank correlation (ρ) was used to examine bivariate relationships between each of the included predictors [24]. Any highly correlated variables (ρ > 0.8) were modeled separately, with only the variable making the most significant contribution to the model (based on the BIC) being retained [24]. Finally, the variables included in the final model were used to build a negative binomial (NB) model and the Vuong test was used to compare the NB and ZINB models with the null hypothesis that the two models were equivalent and the alternative hypothesis that the ZINB model was superior.

### Qualitative Analysis

Thematic and narrative qualitative analysis [25] was used in order to: 1) provide insight into the results of the quantitative analysis, 2) capture information not captured by the quantitative aspects of the tool, 3) provide increased resolution for environmental data by allowing the observer to note special features within the block (vs. the quantitative aspects of the tool, which pertained to the block as a whole), and 5) aid in triangulation for the purposes of identifying the environmental characteristics most significantly associated with rat abundance.

Data included in the qualitative analysis included written notes recorded in the environmental observational tool, as well as field notes recorded during a two-day long debrief at the end of the project. This debriefing involved re-visiting each block included in the study area and discussing observer opinions of the block environment and its relationship to rat abundance.

Handwritten notes were transcribed into Microsoft Excel (Microsoft, USA) and labeled with block number and block trap success. Emergent thematic analyses of early observations were discussed among the two observers and served to inform the focus of subsequent observations, as well as ongoing analyses. The coding framework employed a priori codes derived from the quantitative observational tool, as well as emergent codes based upon ongoing observational work. All qualitative data were reviewed, and text segments related to each individual code were categorized/classified (to identify commonalities among blocks and special features within a block). Subsequent coding passes were used to refine and expand code categories and to identify instances of negative evidence.

## Results

### Quantitative Findings

There was no significant autocorrelation of rat abundance among blocks (p  =  0.14) based on Global Moran's I, supporting our assertion that the city blocks could be treated as independent units. Bivariate relationships between the outcome and each of the 37 potential predictors are presented in Table S1. The final ZINB model included one variable in the zero-inflation portion of the model and six variables in the negative binomial portion of the model (Table 1). None of the variables included in the final model were highly correlated (ρ < 0.45 for all comparisons) and the Vuong test indicated that the ZINB model was superior to the NB model (P  =  0.03).

**Table 1:** Relationship between features of the urban environment and relative abundance of Norway rats (*Rattus norvegicus*).

Variable #	Variable description	β	SE	P-Value	exp (β)	95% CI
***Count Model (negative binomial with log link)***						
10	Proportion of block occupied by **abandoned** parcels	0.70	0.32	0.027	2.02	1.08–3.77
14	Proportion of block occupied by **single family houses**	−0.67	0.25	0.007	0.51	0.31–0.83
19	Proportion of block occupied by **housing over commercial**	0.65	0.21	0.002	1.91	1.27–2.87
c26t30	General **building condition**	−2.01	0.58	0.001	0.13	0.04–0.42
48	Amount of **garbage/trash/junk/litter**	0.83	0.28	0.003	2.29	1.33–3.93
58	Amount of **transport**	−0.99	0.24	< 0.001	0.37	0.23–0.59
***Zero-Inflation Model (binomial with logit link)***						
5	Proportion of block occupied by institutional parcels	−3.42	1.44	0.012	0.03	0.002–0.55

Zero-inflated negative binomial model for the number of rats trapped in a city block offset by the natural logarithm of the trap effort in that block.

The final model indicated that as the proportion of a block occupied by institutional parcels (i.e., those dedicated to not-for-profit services, such as churches, outreach centres, etc.) increased, the odds of not trapping any rats decreased (Table 1). As the proportion of a block occupied by single-family homes increased, the trap success rate decreased. In contrast, as the proportion of the block occupied by housing over commercial and by abandoned parcels (i.e., those with structures that were previously used but are now vacant and not undergoing construction or demolition) increased, the trap success rate increased. As general building condition increased, trap success rate decreased, whereas trap success rate increased as the as the amount of garbage/trash/junk/litter in the alley increased. Finally, as the use of an alley as a transportation corridor increased, trap success rate decreased.

### Qualitative Findings

#### Relationship between rat abundance and land use

Observers often reported trapping rats around abandoned buildings, demolished buildings, and vacant lots. In one block, a large parcel filled with debris from a demolished building was observed to be heavily infested with rats.

Blocks with low trap success tended to be described as being primarily residential (particularly single family homes) or primarily industrial (including industrial sites producing food), while blocks with high trap success tended to be described as being more mixed-use. Observers noted that there was no clear association between the presence of facilities dealing with food (e.g., restaurants, groceries, etc.) and rat presence/abundance.

#### Relationship between rat abundance and socioeconomic status of a block

Observers reported that rats were much more abundant in the impoverished DTES neighborhood than the adjacent more affluent Gastown neighborhood. Observers also reported that blocks composed primarily of single-family homes appeared to be much more affluent than other residential areas, and that many of the low-rise apartments and housing over commercial was low income housing and single-room occupancy hotels (SROs). Low-income apartments, SROs, and outreach centres for impoverished community members were more commonly reported in blocks with high trap success. Also, within a block, observers often reported trapping rats near SROs.

#### Relationship between rat abundance and property/building condition

Buildings in disrepair were much more commonly reported in blocks with high trap success vs. blocks with low trap success. Also, within a block, observers often reported trapping rats around buildings in disrepair. Conversely, buildings and properties were much more commonly reported to be in good to excellent condition in blocks with low trap success. However, observers reported trapping rats around buildings in good repair and vice versa. Observers noted that all aspects of a block had to be assessed to adequately evaluate repair. In some blocks, the street face appeared to be in good repair while the alley face was in very poor repair.

#### Relationship between rat abundance and green space

Well maintained green space and food gardens were more often reported in blocks with low trap success. Unkempt green space was reported in blocks with high and low trap success. However, within a block, observers often reported trapping rats near unkempt green space.

#### Relationship between rat abundance and waste

Observers reported debris, strewn garbage, and overflowing garbage bins much more commonly in blocks with high trap success. Within a block, observers also reported trapping rats in close proximity to areas with accumulated refuse and around dumpsters. Blocks with low trap success were most commonly described as clean with very little garbage. However, observers also noted that some blocks with high trap success were relatively clean. Observers commented that rats were not caught around organic waste bins or compost piles.

#### Relationship between rat abundance and condition of the alley surface

Blocks with high trap success tended to be described as having pavement in disrepair, cracked pavement, unpaved areas, and rat holes, while blocks with low trap success tended to be described as being well paved and having pavement in good repair. Within an alley, observers reported trapping rats around areas where the pavement was in disrepair and/or deeply cracked, around unpaved areas, and around rat holes, regardless of the condition of the rest of the alley. However, some blocks with high trap success were reported to have good alley surface condition and vice versa.

#### Sources of harborage for rats

Observers suggested that most rats likely live in buildings or in underground burrows (rat holes were commonly seen in blocks with high trap success). However, observers also reported trapping rats near areas with stored wood pallets, plastic barrels, and other containers. In four blocks, observers reported frequently catching rats near rat corridors (i.e., access points, such as gaps between buildings, which connected the alley face to deeper aspects of the block).

#### Effect of human activity on rat presence

Heavy use of alleyway by people (particularly people loitering) was reported in blocks with high and low trap success, but was more commonly reported in blocks with high trap success.

Observers noted that the alleyways more heavily used for loitering tended to be dirtier (i.e., have more accumulated refuse).

## Discussion

Using quantitative and qualitative analysis, we were able to identify a variety of environmental factors associated with rat abundance. We also found that once observers were comfortable with the use of the tool, its implementation was relatively efficient and straight forward.

As has been found in previous studies using SSO, the combination of quantitative and qualitative analysis was particularly beneficial. Not only did the qualitative data support and elucidate the quantitative results, these data also provided additional insight into environmental variations within a block, and drew our attention to factors not well captured by the quantitative aspect of the tool (e.g., the apparent association between rat abundance and poverty), as well as inconsistencies in the relationship between certain environmental factors and relative rat abundance.

This study was able to identify a number of factors associated with relative rat abundance that have not been studied and/or well characterized previously (e.g., specific land use, residential density, and building disrepair). Several of our findings, however, are in agreement with previous research on the relationship between environmental factors and rat abundance. For example, studies have been able to show that the presence of exposed garbage is a strong predictor/promoter of rat infestations [6], [13]. Our study also found that rats were relatively less common in blocks where the land use was predominantly industrial and single-family residential, and this is consistent with the findings of Cavia et al. (2009), who also found that Norway rats were uncommon in industrial and residential neighborhoods.

Other studies have also indicated that factors associated with poverty can promote rat infestations, likely due to a combination of factors including infrastructural disrepair and decreased environmental hygiene, which combine to create ideal urban rat habitat [3], [5]. Poverty might also impair an individual's or community's ability to prevent or eliminate infestations (e.g., through neighborhood rehabilitation or employing pest control professionals). In our study, there were several variables that, on first glance, are difficult to intuitively link to rat abundance, including proportion of block occupied by institutional parcels and housing over commercial. However, upon further examination, and in light of the qualitative data, it became clear that much of the housing over commercial was low-income housing, including single room occupancy hotel rooms (SROs). Similarly, institutional parcels in the area are primarily composed of outreach centers for impoverished members of the community. Observers specifically noted that rats were often trapped around SROs and that blocks with high trap success tended to have more SROs, low-income housing, and outreach centers. Observers also noted that far more rats were trapped in the impoverished DTES compared to the adjacent, more affluent Gastown district, and that blocks composed primarily of single-family residences were also more affluent than the rest of the DTES. Factors potentially associated with poverty were associated with trap success independent of variables accounting for infrastructural disrepair and accumulation of waste. This suggests that there are likely one or more environmental variables associated with poverty that were not accounted for in this study.

It is interesting to note that season did not appear to have a significant relationship with rat presence or abundance. Indeed, there has been considerable debate in the literature regarding the impact of season on rats in urban ecosystems [1]. The absence of seasonal variation in urban rat population size might not be surprising given that urban ecosystems, being largely under anthropogenic control, would seem to be less prone to seasonal fluctuation in resource availability (i.e., the presence of suitable food and harborage) compared to more sylvatic settings. However, given that this study only included one year's worth of data, the true impact of season could not be definitively determined [26].

There were a number of findings in our study that contrast with those found in past research on the subject. For example, green space has been reported to be a strong promoter of rat infestations [4], [6]. In particular, a study by Traweger et al. (2008) found that rats were most abundant in more natural areas with abundant vegetation, and that trap success was very low in areas with little vegetation, as well as heavily built-up areas, and areas frequented by humans. Our study found that there was no relationship between amount of green space and rat abundance, that rats were very common in built up areas, and that human activity (in the form of loitering) appeared to have a negligible to positive relationship with rat abundance. Increased use of an alley as a transport corridor did appear to have a negative association with the number of rats trapped. However, it is difficult to determine if this because rats avoid areas of increased activity or if alleys used for transport tended to be in better condition or in areas with other features that deter rat infestations. Although Traweger et al. (2008) speculate that natural areas are essential to providing appropriate rat habitat, the results of our study indicate that Norway rats can thrive even in the most urbanized of settings.

Interestingly, the qualitative data also indicated the presence of conflicts within our own study. For example, although strewn garbage seemed to be strongly associated with rat abundance in general, there were some alleys and/or alley segments where rats were caught that appeared relatively clean. Similarly, although many rats were caught in alleys with unpaved areas, cracked pavement, and/or rat holes, some alleys with robust rat populations appeared to have relatively good pavement condition. These conflicts, in combination with the variety of environmental factors that were associated with trap success in our study, suggest that the relationship between rat abundance and the environment is a function of a complex interaction of factors. In other words, there is not one single environmental factor that is a necessary determinant of rat abundance. Rather, a variety of different factors can combine to create suitable rat habitat.

Despite the success of the methodology, this study had several limitations. The relatively low number of observations (n  =  43) limited the number of variables that could be included in the model. Future studies should seek to increase power (i.e., the number of blocks included) in order to better investigate the full suite of variables that might predict rat abundance. With regard to our trapping methodology, only rats accessing the back alley were accounted for in our measure of rat abundance. Although this ‘alley trapping’ approach is consistent with previous trapping studies of urban rats [7], [8], it is possible that some rats could reside entirely indoors and/or not access the alley. This may particularly be the case for black rats, as it has been suggested that these species prefer to reside in human structures [1]. Indeed, in this study, it could not be determined if the low number of black rats was a result of the trapping methodology, or whether this species is truly rare within this urban area. Ideally, future studies should account for all indoor and outdoor rat populations within a block. However, as this would require intensive trapping on private property, it may not be a feasible proposition. Finally, it should be noted that while many factors have a clear direct relationship with rat abundance (e.g., presence of exposed garbage); other factors (e.g., land use) likely represent some underlying and unmeasured factor(s) that influence rat populations. Although these environmental ‘proxies’ may be valuable for predicting rat abundance, they are less useful for developing intervention strategies. After all, it is not reasonable to change the land use in a block to prevent rat infestations. It may therefore be useful for future studies to focus on and dissect the relationship between rat abundance and factors like land use and poverty.

Overall, this tool proved to be an efficient and effective way to examine the effect of urban environmental factors on relative rat abundance. In addition to their effects of rat abundance, environmental factors are also known to influence pathogen prevalence in rat populations [2]. In the future, we hope to determine whether this tool could also be used to identify features of the urban environment associated with the prevalence of zoonotic microbes in rats.

## Supporting Information

Appendix S1 **A systematic environmental observation tool to predict relative rat abundance in urban centres.**
(PDF)

Appendix S2 **Guidance document for the systematic environmental observation tool.**
(PDF)

Table S1 **Quantitative predictors generated from the environmental observation tool.**
(DOCX)

Table S2 **Bivariate relationships between features of the urban environment and relative abundance of Norway rats (** ***Rattus norvegicus*** **).** Zero-inflated negative binomial model for the number of rats trapped in a city block offset by the natural logarithm of the trap effort in that block.
(DOCX)
