# Supplementary material for: A Mixed Methods Approach to Exploring the Relationship between Norway Rat (Rattus norvegicus) Abundance and Features of the Urban Environment in an Inner-City Neighborhood of Vancouver, Canada
Source: PLoS One. 2014 May 15;9(5):e97776. doi: 10.1371/journal.pone.0097776 (PMC4022650; doi:10.1371/journal.pone.0097776)
Supplement: Appendix S1 — A systematic environmental observation tool to predict relative rat abundance in urban centres. (PDF) [file pone.0097776.s001.pdf]

VANCOUVER RAT PROJECT
ENVIRONMENTAL OBSERVATION TOOL

Date: ________________________________
Block Number:__________________________
Block Location: _________________________
____

All block evaluations are to take place during working hours (8:00 am to 4:00 pm, Monday – Friday). For features showing temporal variation (e.g., human activity) night time and week-end characteristics will not be captured.

Block evaluations should take place as close to the time of active trapping as possible. This will ensure that the environmental characteristics recorded reflect those present at the time of trapping.

Unless otherwise stated, consider the block as a whole (including any and all features of that block visible from any block or alley face or that can be derived from aerial maps).

Land Use Characteristics

Parcel Allotment

The following questions ask you to classify parcels as a whole according to their predominant usage.

Residential parcels include any parcel dedicated to be a permanent residence (e.g., houses, townhomes, etc.). Commercial parcels include those dedicated to retail or business (e.g., stores, restaurants, etc.). Industrial parcels include those dedicated to the production or processing of products (i.e., factories, warehouses, etc.). Institutional parcels include those dedicated to not-for-profit services (i.e., churches, outreach centers,etc.). Green space parcels include those dedicated solely to green space (i.e., gardens, parks, etc.). Parcels that include green space as well as structures should be assigned the land use of the structure. Vacant/undeveloped parcels refer to plots of land which are clearly not in use as indicated by lack of a structure on the property or lack of land development. Parcels under demolition include those containing buildings that are in the process of being demolished (parcels containing the remains of long demolished buildings should be considered vacant/undeveloped). Parcels that are under construction include those with buildings undergoing construction or major renovation and that do not contain any occupants. Abandoned parcels include those with structures that were previously used but are now vacant and not undergoing construction or demolition (do not include undeveloped lots, which have no structures). Open parcels include those that do not contain any buildings but that are not vacant/undeveloped, demolished, or green spaces (e.g., parking lots). Code mixed only if a predominant form cannot be determined (e.g. a street has 3 residences and 3 commercial stores). Code other if none of the above categories apply.

1. How would you characterize the **land use** on this block? **(Code the predominant form)**

Primarily residential..............................1
Primarily commercial….........................2
Primarily industrial …...........................3
Primarily institutional ……….................4
Primarily green space……….................5
Primarily vacant/undeveloped lots........6
Primarily under demolition ……..........7
Primarily under construction………......8
Primarily abandoned…………………..9
Primarily open spaces………………..10
Mixed ………………........................11
Other ……………………….............12

If you stated 'other' please specify:
____
____
____
____

Answer q. 2-12 according to the total block area occupied by parcels corresponding to each of the following land uses.

2. Approximately what proportion of the block is occupied by **residential** parcels?

None ……………...........................0
Less than ¼.......................................1
Between ¼ and ½.............................2
Between ½ and ¾ 3
More than ¾ 4

3. Approximately what proportion of the block is occupied by **commercial** parcels?

None ………….............................0
Less than ¼......................................1
Between ¼ and ½..............................2
Between ½ and ¾ 3
More than ¾ 4

4. Approximately what proportion of the block is occupied by **industrial** parcels?

None ………….............................0
Less than ¼......................................1
Between ¼ and ½..............................2
Between ½ and ¾ 3
More than ¾ 4

5. Approximately what proportion of the block is occupied by **institutional** parcels?

None ………….............................0
Less than ¼......................................1
Between ¼ and ½..............................2
Between ½ and ¾ 3
More than ¾ 4

6. Approximately what proportion of the block is occupied by **green space** parcels?

None ………….............................0
Less than ¼......................................1
Between ¼ and ½..............................2
Between ½ and ¾ 3
More than ¾ 4

7. Approximately what proportion of the block is occupied by **vacant/undeveloped** parcels?

None ………….............................0
Less than ¼......................................1
Between ¼ and ½..............................2
Between ½ and ¾ 3
More than ¾ 4

8. Approximately what proportion of the block is occupied by parcels **under demolition**?

None ………….............................0
Less than ¼......................................1
Between ¼ and ½..............................2
Between ½ and ¾ 3
More than ¾ 4

9. Approximately what proportion of the block is occupied by parcels **under construction**?

None ………….............................0
Less than ¼......................................1
Between ¼ and ½..............................2
Between ½ and ¾ 3
More than ¾ 4

10. Approximately what proportion of the block is occupied by **abandoned** parcels?

None ………….............................0
Less than ¼......................................1
Between ¼ and ½..............................2
Between ½ and ¾ 3
More than ¾ 4

11. Approximately what proportion of the block is occupied by **open** parcels?

None ………….............................0
Less than ¼......................................1
Between ¼ and ½..............................2
Between ½ and ¾ 3
More than ¾ 4

12. Approximately what proportion of the block is occupied by **other** land uses?

None ………….............................0
Less than ¼......................................1
Between ¼ and ½..............................2
Between ½ and ¾ 3
More than ¾ 4

Residential Density

Questions 13-18 seek to quantify residential density.

13. How would you characterize the **predominant housing type** on this block?

No residential units …………...............0
Single-family houses................................1
Duplex/Rowhouses...................................2
Low rise apartment or condominium buildings (1-4 floors)….……………...........3
Mid-rise apartment or condominium buildings (4-10 floors)..............................5
High-rise apartment or condominium buildings (> 10 floors)……………………...6
Housing units over commercial store fronts. 7
Mixed ……………………………………8

Code _mixed_ *only if a predominant form cannot be determined (e.g. a street has 3 single family units and 3 duplexes).*

If you stated ‘mixed’ please specify:
____
____
____
____

Answer the following questions according to the _total area_ *occupied by the* _structures_ *of interest (vs. the parcels as a whole).*

14. Approximately what proportion of the block is occupied by **single family houses**?

None …………….........................0
Less than ¼......................................1
Between ¼ and ½.............................2
Between ½ and ¾ 3
More than ¾ 4

15. Approximately what proportion of the block is occupied by **duplexes or rowhouses**?

None …………….........................0
Less than ¼......................................1
Between ¼ and ½.............................2
Between ½ and ¾ 3
More than ¾ 4

16. Approximately what proportion of the block is occupied by **low-rise apartment or condominium buildings (1-4 floors)**?

None …………….........................0
Less than ¼......................................1
Between ¼ and ½.............................2
Between ½ and ¾ 3
More than ¾ 4

17. Approximately what proportion of the block is occupied by **mid-rise apartment or condominium buildings (5-10 floors)**?

None …………….........................0
Less than ¼......................................1
Between ¼ and ½.............................2
Between ½ and ¾ 3
More than ¾ 4

18. Approximately what proportion of the block is occupied by **high-rise apartment or condominium buildings (>10 floors)**?

None …………….........................0
Less than ¼......................................1
Between ¼ and ½.............................2
Between ½ and ¾ 3
More than ¾ 4

19. Approximately proportion of the block is occupied by **housing units over commercial storefronts?**

None …………….........................0
Less than ¼......................................1
Between ¼ and ½.............................2
Between ½ and ¾ 3
More than ¾ 4

Food-Associated Non-Residential Land Use

The following questions (q. 20-24) seek to identify and quantify the number of establishments _producing, storing, processing, selling, and/or providing food_ *(not including residential buildings). ‘Other’ buildings providing food include those not covered by the other categories but that can be reasonably expected to provide food to patrons (i.e., outreach centers, benevolence societies, etc.)*

For buildings with different usages on different levels (i.e., non-residential units with housing above), the area of that building should be included in the following estimates of non-residential land use.

Answer the following questions according to the _total area_ *occupied by the* _structures_ *of interest (vs. the parcels as a whole).*

20. Approximately proportion of the block is occupied by non-residential **buildings not associated with food**?

None …………….........................0
Less than ¼......................................1
Between ¼ and ½.............................2
Between ½ and ¾ 3
More than ¾ 4

21. Approximately proportion of the block is occupied by **restaurants**?

None …………….............................0
Less than ¼......................................1
Between ¼ and ½..............................2
Between ½ and ¾ 3
More than ¾ 4

22. Approximately proportion of the block is occupied by **groceries or other commercial venues selling food**?

None …………….............................0
Less than ¼......................................1
Between ¼ and ½..............................2
Between ½ and ¾ 3
More than ¾ 4

23. Approximately proportion of the block is occupied by **industrial establishments producing, processing, and/or storing food**?

None …………….............................0
Less than ¼......................................1
Between ¼ and ½..............................2
Between ½ and ¾ 3
More than ¾ 4

24. Approximately proportion of the block is occupied by **'other' establishments associated with food?**

None …………….............................0
Less than ¼......................................1
Between ¼ and ½..............................2
Between ½ and ¾ 3
More than ¾ 4

If you stated 'other' buildings providing food please specify:

____
____
____

Property Condition

Use the following guide to code for building and property conditions.

Extremely poor: Structure looks unfit/unsafe for humans. Significant amount of broken glass, peeling paint, damaged structures (e.g. stairs, walls, etc.). Dirty and unkempt property with little or no apparent regard for upkeep of property. Major overhaul needed to improve property. Abundant refuse and/or severe overgrowth. May include abandoned structures and properties. Poor: Adequate living/workings conditions (albeit poor environment). Moderate amount of broken glass, peeling paint, damaged structures, etc. Considerable amount of work needed to improve property. Moderate amounts of refuse and/or overgrowth. Fair: Passable conditions with some attempt to keep property but moderate success. Some broken windows, peeling paint, damaged structures, etc. Would recommend a moderate amount of repair. Some refuse/overgrowth. Good: In decent working/living condition. Some repairs could be done (mainly for aesthetic reasons). Looks clean and well kept. Excellent: Immaculate/near perfect condition. No repair needed. Shows that extra care and effort has been directed towards upkeep. Mixed condition: Only code for two-grade differences wherein at least 25% of the properties are at one grade and 25% are two grades higher.

Answer q.24-36 based on your general impression of the block as a whole. For building condition, discount any buildings undergoing demolition, construction, or major renovation.

25. What is the general condition of the **buildings**?

None/not applicable………..…………0
Extremely poor………………………..1
Poor..2
Fair...3
Good 4
Excellent..5
Mixed (extreme difference)................6

26. Approximately what proportion of the block is occupied by **buildings in extremely poor condition**?

None …………….............................0
Less than ¼......................................1
Between ¼ and ½..............................2
Between ½ and ¾ 3
More than ¾ 4

27. Approximately what proportion of the block is occupied by **buildings in poor condition**?

None …………….............................0
Less than ¼......................................1
Between ¼ and ½..............................2
Between ½ and ¾ 3
More than ¾ 4

28. Approximately what proportion of the block is occupied by **buildings in fair condition**?

None …………….............................0
Less than ¼.......................................1
Between ¼ and ½..............................2
Between ½ and ¾ 3
More than ¾ 4

29. Approximately what proportion of the block is occupied by **buildings in good condition**?

None …………….............................0
Less than ¼.......................................1
Between ¼ and ½..............................2
Between ½ and ¾ 3
More than ¾ 4

30. Approximately what proportion of the block is occupied by **buildings in excellent condition**?

None …………….............................0
Less than ¼.......................................1
Between ¼ and ½..............................2
Between ½ and ¾ 3
More than ¾ 4

Grounds include <u>any space</u> or parcel within a block that is <u>not occupied by a building</u>. May include open/undeveloped/green space parcels, as well as open spaces in a parcel with structures.

31. What is the general condition of the **grounds?**

None/not applicable...............................0
Poor/deteriorated...................................1
Fair..2
Good..3
Excellent..4
Mixed (extreme difference)...................5

32. Approximately what proportion of the block is occupied by **grounds in extremely poor condition**?

None …………….............................0
Less than ¼.......................................1
Between ¼ and ½..............................2
Between ½ and ¾ 3
More than ¾ 4

33. Approximately what proportion of the block is occupied by **grounds in poor condition**?

None …………….............................0
Less than ¼.......................................1
Between ¼ and ½..............................2
Between ½ and ¾ 3
More than ¾ 4

34. Approximately what proportion of the block is occupied by **grounds in fair condition**?

None …………….............................0
Less than ¼.......................................1
Between ¼ and ½..............................2
Between ½ and ¾ 3
More than ¾ 4

35. Approximately what proportion of the block is occupied by **grounds in good condition**?

None …………….............................0
Less than ¼.......................................1
Between ¼ and ½..............................2
Between ½ and ¾ 3
More than ¾ 4

36. Approximately what proportion of the block is occupied by **grounds in excellent condition**?

None …………….............................0
Less than ¼.......................................1
Between ¼ and ½..............................2
Between ½ and ¾ 3
More than ¾ 4

Green Space Characteristics

For an area to be considered a green space it must: 1) show significant plant growth (i.e., areas with sparse grass or weeds should <u>not</u> be included) and 2) occupy a significant proportion of the property parcel (i.e., very small areas of plant growth in an otherwise build-up parcel should not be included).

Answer the following questions according to the <u>total area</u> occupied by the <u>green spaces</u> of interest (vs. parcels as a whole).

37. Approximately what proportion of the block is occupied by **green space**?

None …………….............................0
Less than ¼.......................................1
Between ¼ and ½..............................2
Between ½ and ¾ 3
More than ¾ 4

Unkempt green space is an area that is overgrown by vegetation and/or does not appear to receive regular maintenance.

38. Approximately what proportion of the block is occupied by **unkempt** green space?

None …………….............................0
Less than ¼.......................................1
Between ¼ and ½..............................2
Between ½ and ¾ 3
More than ¾ 4

Well kept green space is considered to be green space that receives regular maintenance. It may include lawns, non-food gardens, or other landscaped areas.

39. Approximately what proportion of the block is occupied by **well kept** green space?

None …………….............................0
Less than ¼.......................................1
Between ¼ and ½..............................2
Between ½ and ¾ 3
More than ¾ 4

Green space that is in any way geared toward the production of produce should be considered to be a food garden.

40. Approximately what proportion of the block is occupied by **food gardens**?

None …………….............................0
Less than ¼.......................................1
Between ¼ and ½..............................2
Between ½ and ¾ 3
More than ¾ 4

Alley Surface Characteristics

For the following questions consider only the alley surface at the alley face. The alley face considered should include the 1m of land on either side of the alley border.

Use the following definitions for questions 41-44. Only paved alley surfaces should be considered for this question.

Poor: Frequent/severe deep cracking of pavement (cracking sufficient to permit rat burrowing). Alley surface is broken up and in need of serious repair. Fair: Moderate amount of cracked pavement. Cracking is mainly superficial (unlikely to promote rat harborage). Good: Generally even surface with minimal cracking.

41. How would you rate the **general** condition of the **paved alley surfaces** at the alley faces?

Poor..1
Fair...2
Good……………...............................3

42. Approximately what proportion of the alley face is in **poor condition**?

None …………….............................0
Less than ¼.......................................1
Between ¼ and ½..............................2
Between ½ and ¾ 3
More than ¾ 4

43. Approximately what proportion of the alley face is in **fair condition**?

None …………….............................0
Less than ¼.......................................1
Between ¼ and ½..............................2
Between ½ and ¾ 3
More than ¾ 4

44. Approximately what proportion of the alley face is in **good condition**?

None …………….............................0
Less than ¼.......................................1
Between ¼ and ½..............................2
Between ½ and ¾ 3
More than ¾ 4

Alley surface not covered in concrete or asphalt is considered unpaved. If there is any unpaved surface within 1 m of the alley border (regardless of total

depth) then that part of the alley face should be considered unpaved. Deeply cracked pavement should still be considered to be paved.

45. Approximately what proportion of the alley face is bordered by some sort of **non-paved surface**?

None …………….............................0
Less than ¼.......................................1
Between ¼ and ½..............................2
Between ½ and ¾ 3
More than ¾ 4

A rat hole is considered to be any hole at the alley face that appears to have been created by a rat or that would reasonably permit the passage of an adult rat.

46. How many **rat holes** are there are at the alley face: ___________

Rat corridors are spaces between buildings that may allow rats to access the alley face from deeper within the property

47. How many **rat corridors** are there at the alley face:______________

Presence of Waste

For the following questions consider only waste present near the alley face on an average day. The alley face considered should include the 5m of land on either side of the alley border.

A little waste is when there is a very low volume of waste or the waste is confined to a small area. A lot of waste when there is a high volume of waste and/or waste is present throughout a large proportion of the alley. Some waste is somewhere between a little and a lot.

For question 48, garbage, litter, and junk include any organic or inorganic materials that have been discarded in places other than a designated receptacle. Open compost piles may be included.

48. Is there **garbage, trash, junk, or litter**?

None 0
A little..1
Some..2
A lot...3

49. Is there **garbage overflowing from receptacles** in the alley?

None 0
A little..1
Some..2
A lot...3

How many of each of the following types of receptacles are there?

50. Commercial garbage_________
51. Private garbage_____________
52. Commercial recycling_________
53. Private recycling_____________
54. Commercial organic_____________
55. Private organic______________

56. Are there any obvious **strong odors** anywhere in the alley face (urine stench, rotting garbage, etc.)?

No 0
Yes..1

Alley Usage

Alleys may be used as transportation corridors (e.g., for foot or vehicular traffic, for moving goods into and out of buildings, etc.). Note that transportation may include transport of people, vehicles, or goods. Alleyways may also be used for loitering. Loitering is considered to be any non-transportation-related activity (i.e., socializing, resting, using drugs, etc.).

Use should be judged holistically through several morning and afternoon visitations during the trapping period. Use may also be inferred through signs of human presence (e.g., left over belongings or refuse, drug paraphernalia, etc.).

An alleyway is lightly used if very few people are seen and/or if people are seen infrequently. An alleyway is heavily used if groups of people are seen using the alley and/or if people are very frequently seen in the alley. A moderately used alley is somewhere between a lightly a heavily used one.

57. How heavily is this alleyway used by people **loitering**?

Not used..0
Lightly used…..1
Moderately used…..................................2
Heavily used...3

58. How heavily is this alleyway used as a **transportation corridor**?

Not used..0
Lightly used…..1
Moderately used…..................................2
Heavily used..3

General Comments
