# Supplementary material for: A Mixed Methods Approach to Exploring the Relationship between Norway Rat (Rattus norvegicus) Abundance and Features of the Urban Environment in an Inner-City Neighborhood of Vancouver, Canada
Source: PLoS One. 2014 May 15;9(5):e97776. doi: 10.1371/journal.pone.0097776 (PMC4022650; doi:10.1371/journal.pone.0097776)

THE ENVIRONMENTAL OBSERVATION TOOL PHOTO LIBRARY

Vancouver Rat Project

Dr. Chelsea G. Himsworth, Alice Feng, & Kirbee Parsons

Explanation

- This presentation provides
  - Photo examples for different rating systems and subjects used in the Vancouver Rat Project Environmental Observation Tool
  - The following ratings are covered:
    - Building Condition
    - Grounds Condition
    - Green Space Characteristics
    - Presence of Waste
    - Paved Surface Condition
  - Miscellaneous Subjects:
    - Garbage Receptacles
    - Rat Holes
    - Rat Corridors

Building Condition

- Rates the general state of repair/disrepair of buildings

Building Condition

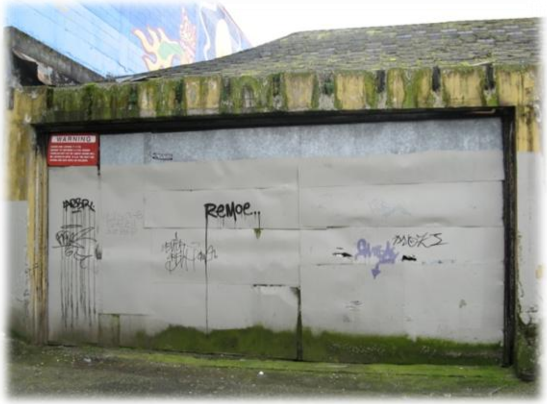

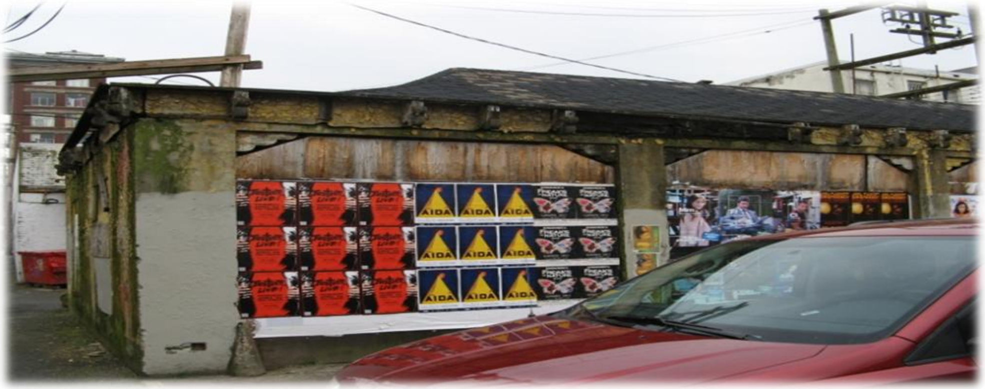

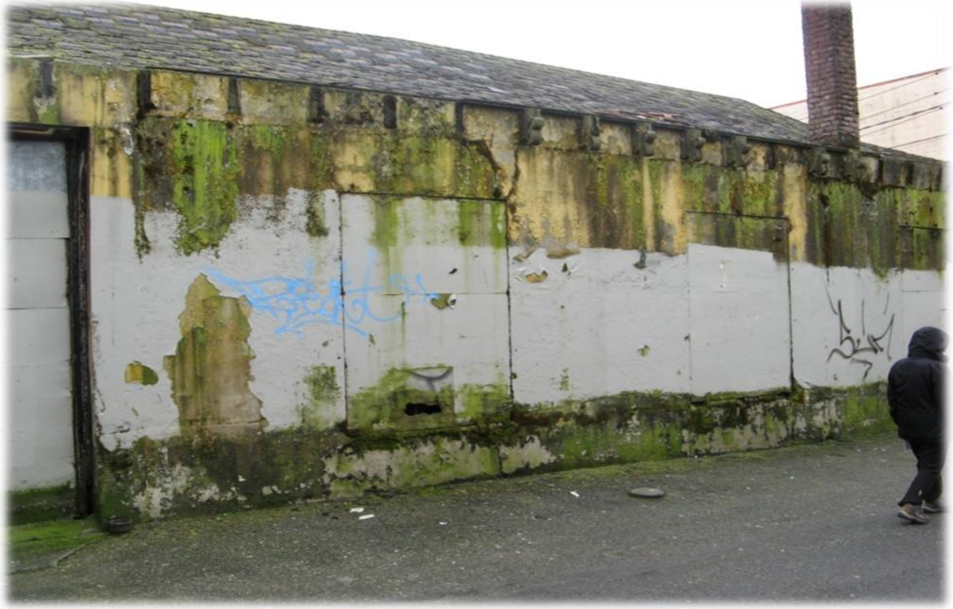

<u>Extremely poor</u>: Structure looks unfit/unsafe for humans. Significant amount of broken glass, peeling paint, damaged structures (e.g. stairs, walls, etc.). May include abandoned structures
and properties.

Building Condition

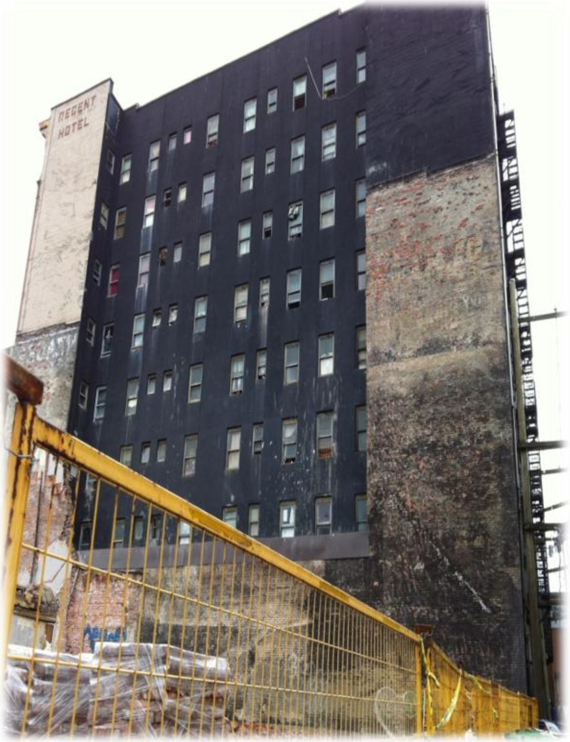

<u>Poor</u>: Adequate living/workings conditions (albeit poor environment). Moderate amount of broken glass, peeling paint, damaged structures, etc. Considerable amount of work needed to improve property.

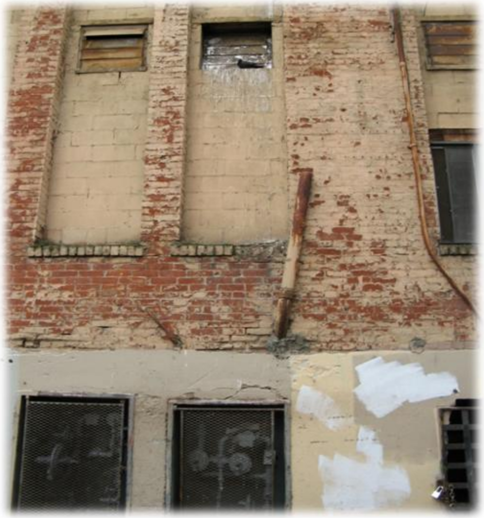

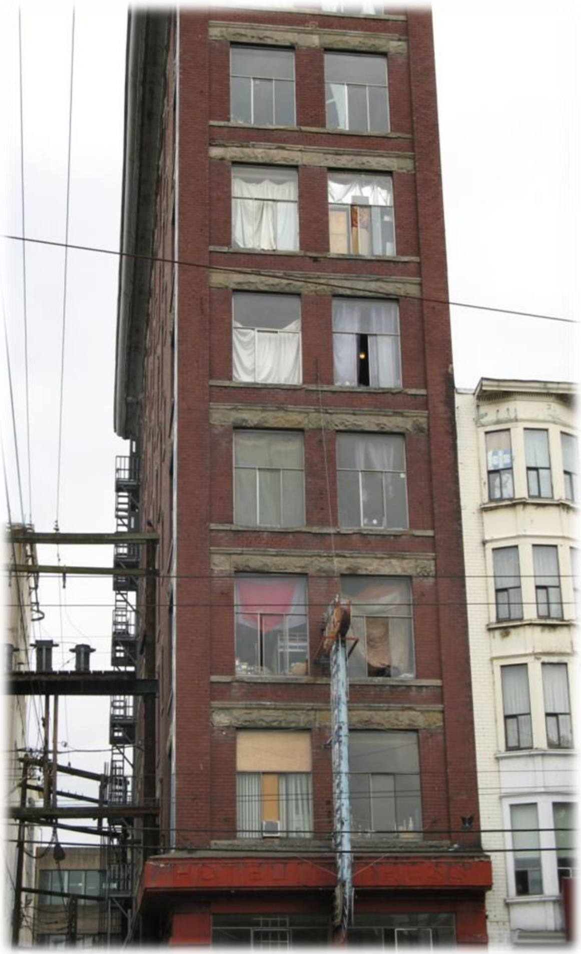

Building Condition

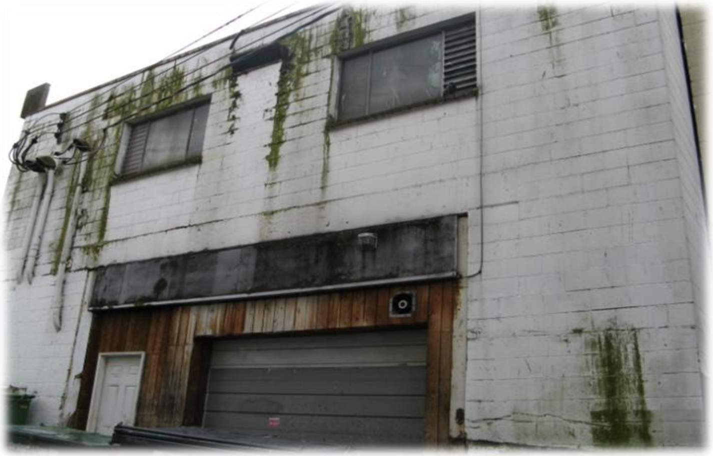

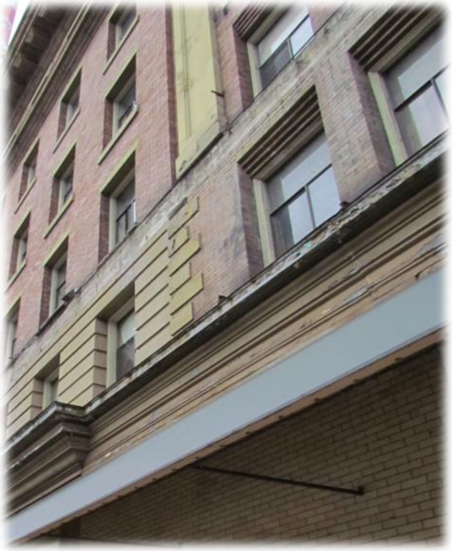

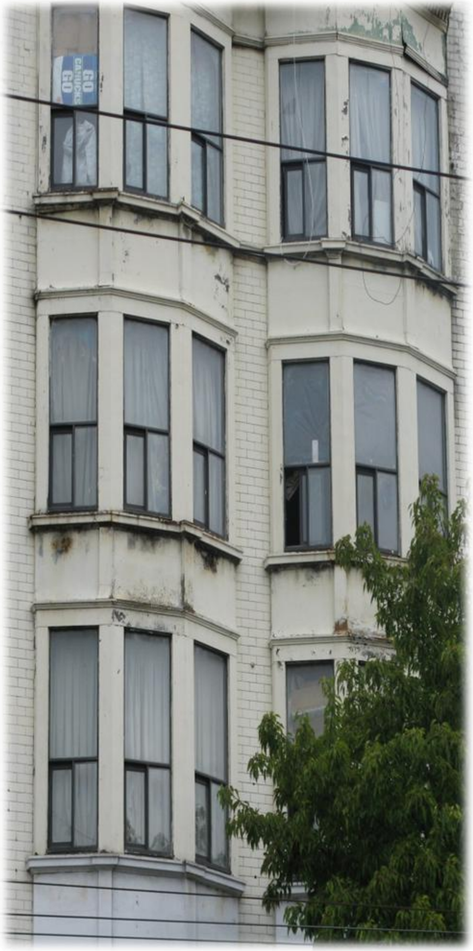

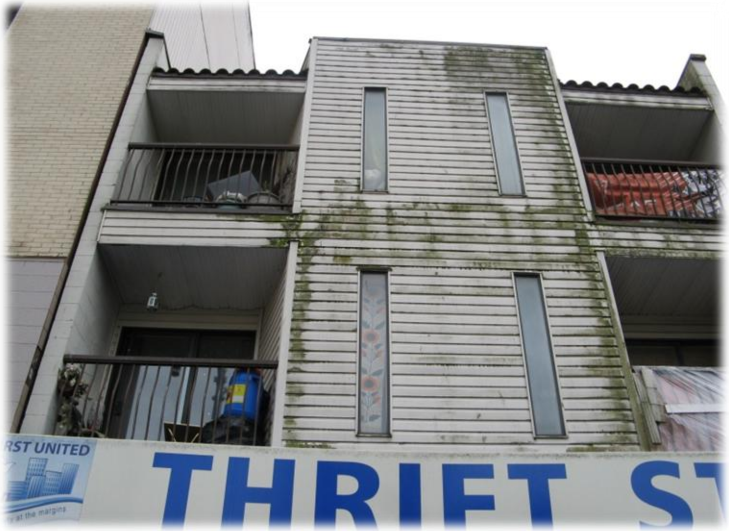

Fair: Passable conditions with some attempt to keep property but moderate success. Some broken windows, peeling paint, damaged structures, etc. Would recommend a moderate amount of repair. Some refuse/overgrowth.

Building Condition

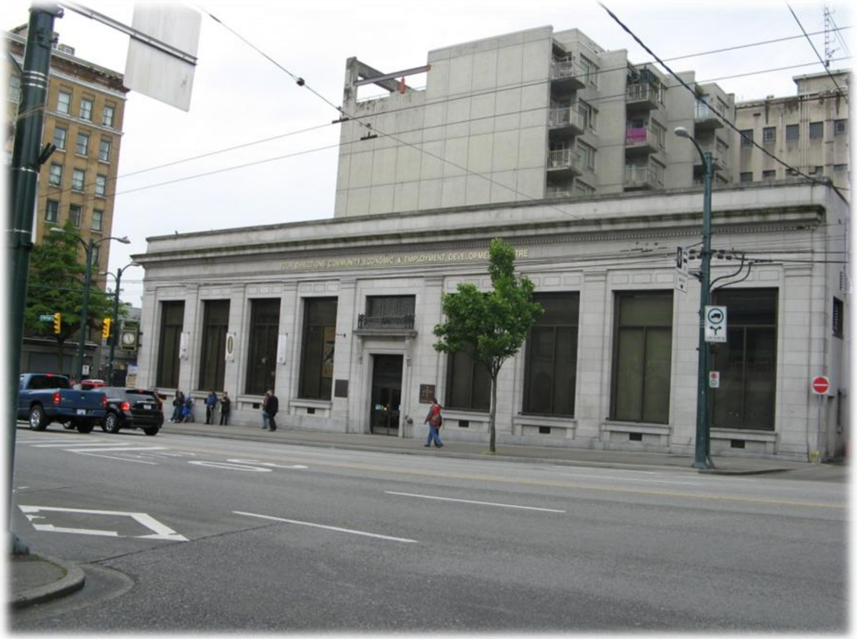

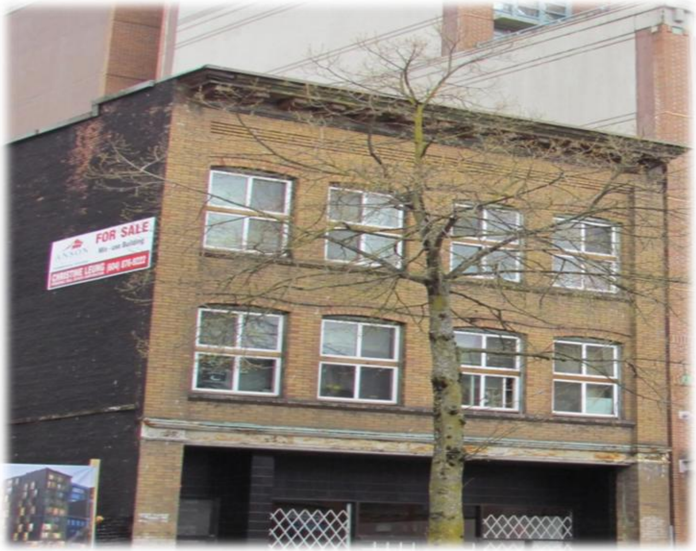

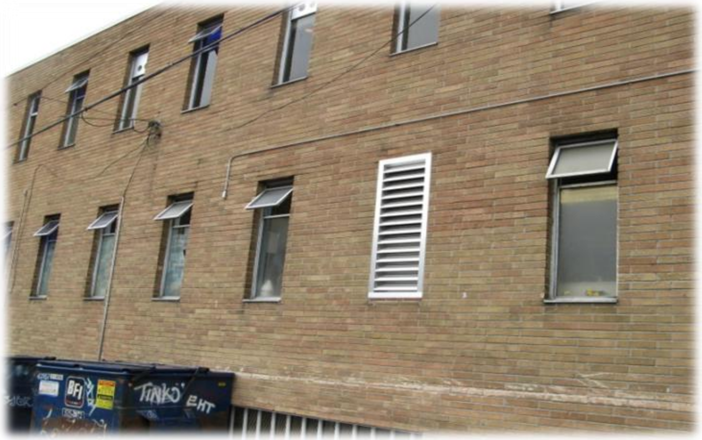

<u>Good</u>: In decent working/living condition. Some repairs could be done (mainly for aesthetic reasons). Looks clean and well kept.

Building Condition

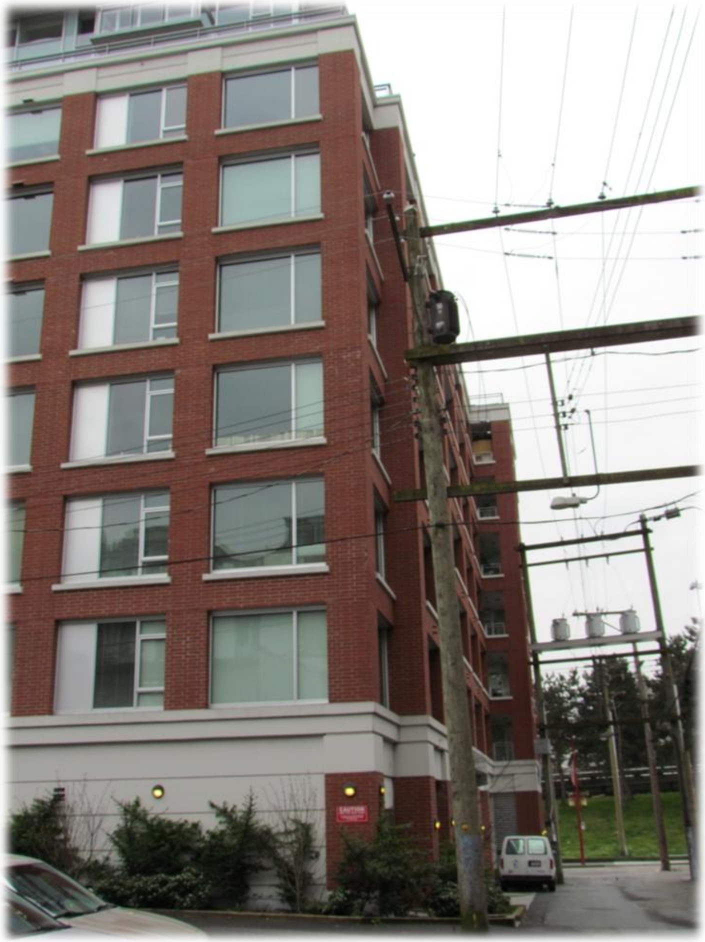

__Excellent__: Immaculate/near perfect condition. No repair needed. Shows that extra care and effort has been directed towards upkeep.

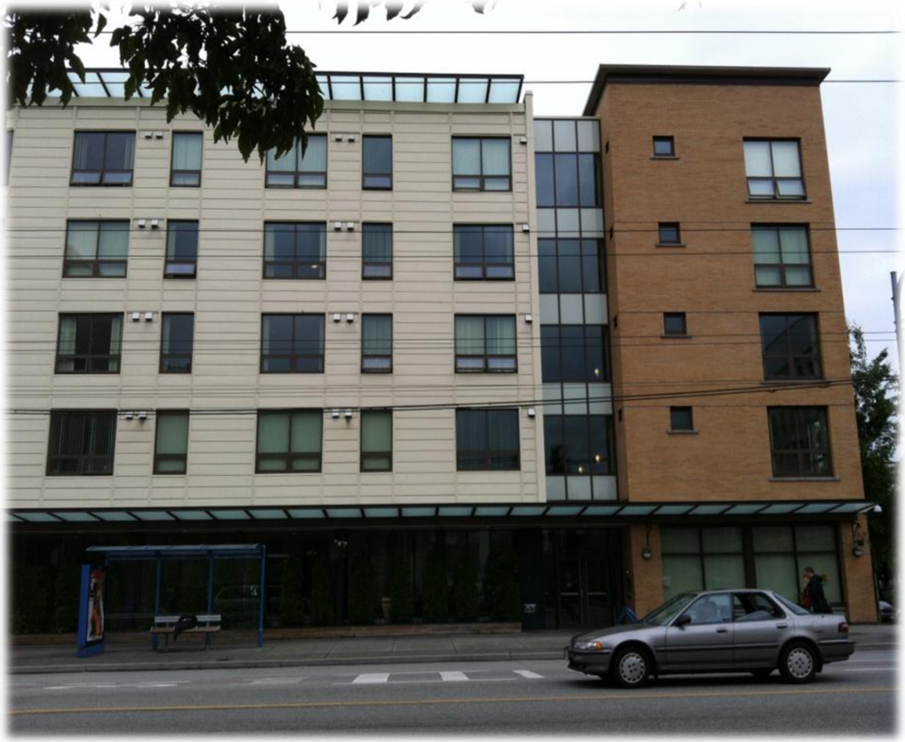

Grounds condition

- Grounds are defined as any space or parcel within a block that is not occupied by a building
  - Includes open, undeveloped or green space parcels, as well as open spaces in a parcel with structures
- Rated for the general state of upkeep of a property (not including building structures)

Grounds Condition

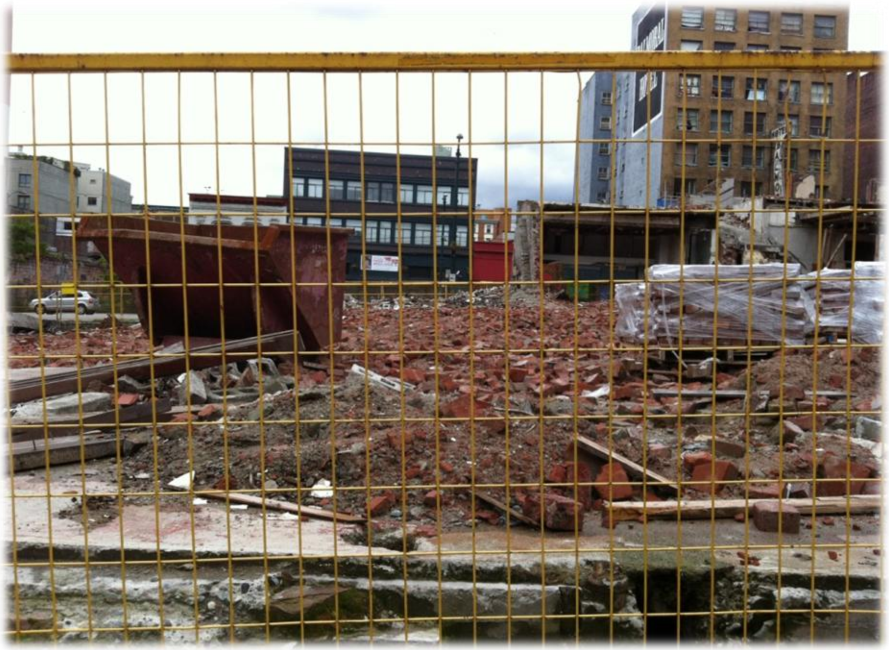

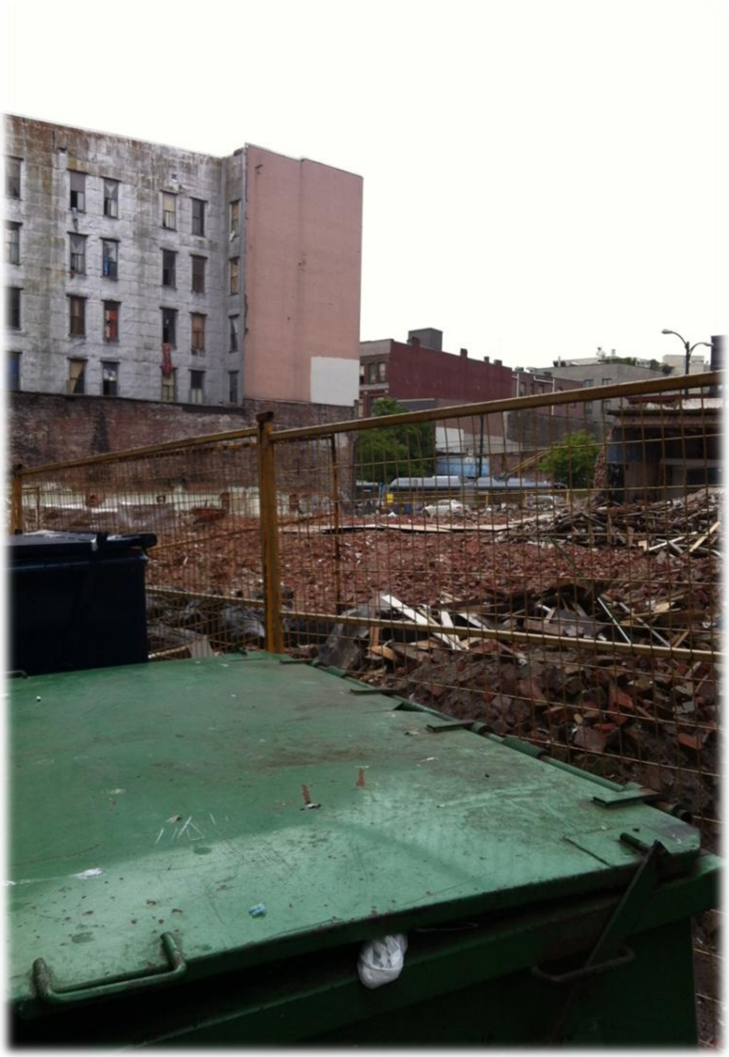

Extremely Poor: Dirty and unkempt property with little or no apparent regard for upkeep of property. Major overhaul needed to improve property.

Grounds Condition

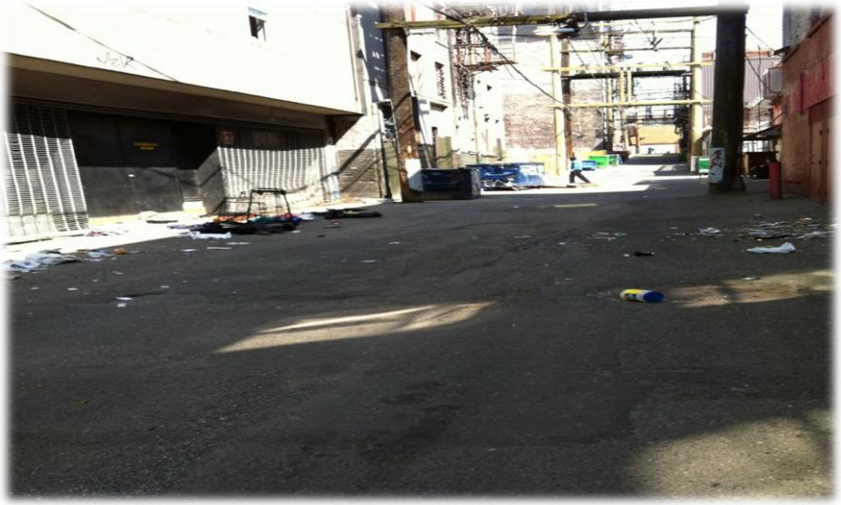

<u>Poor:</u> *Considerable amount of work needed to improve property. Moderate amounts of refuse and/or overgrowth.*

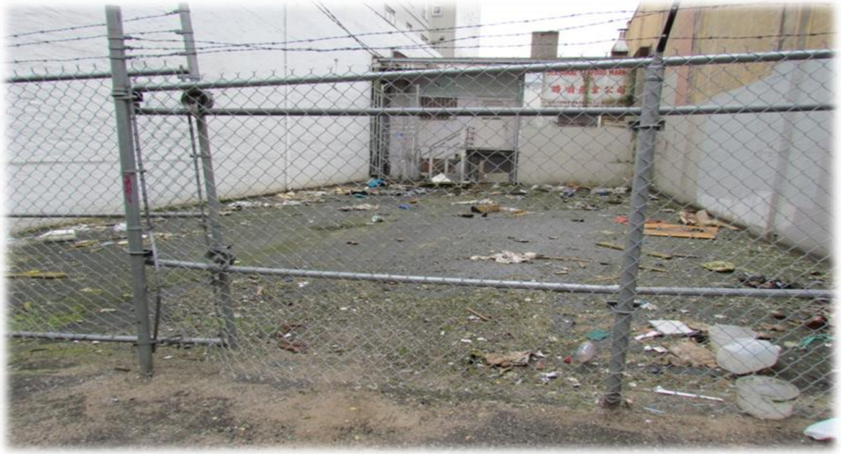

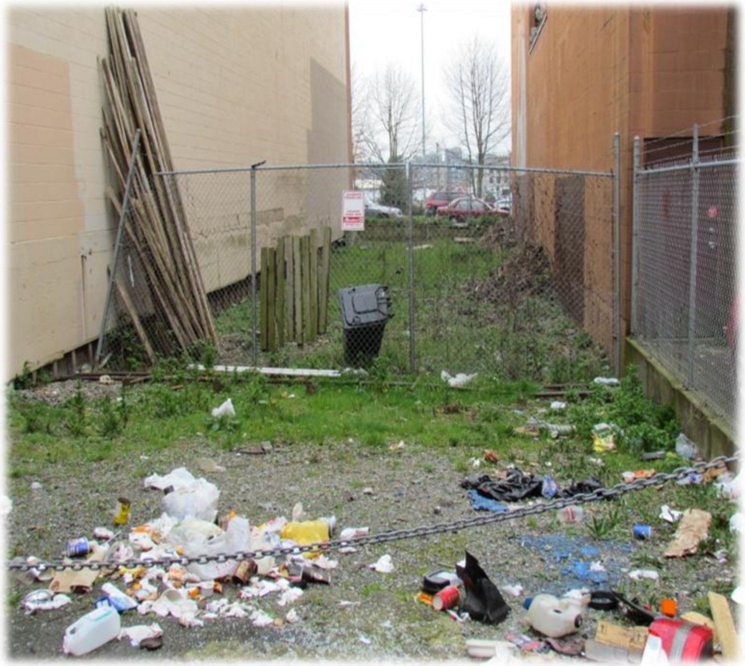

Grounds Condition

<u>Fair:</u> Passable conditions with some attempt to keep property but moderate success. Some refuse/overgrowth.

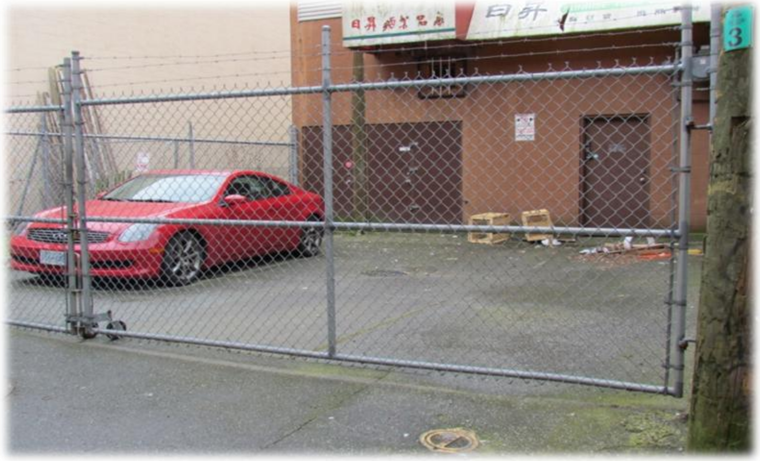

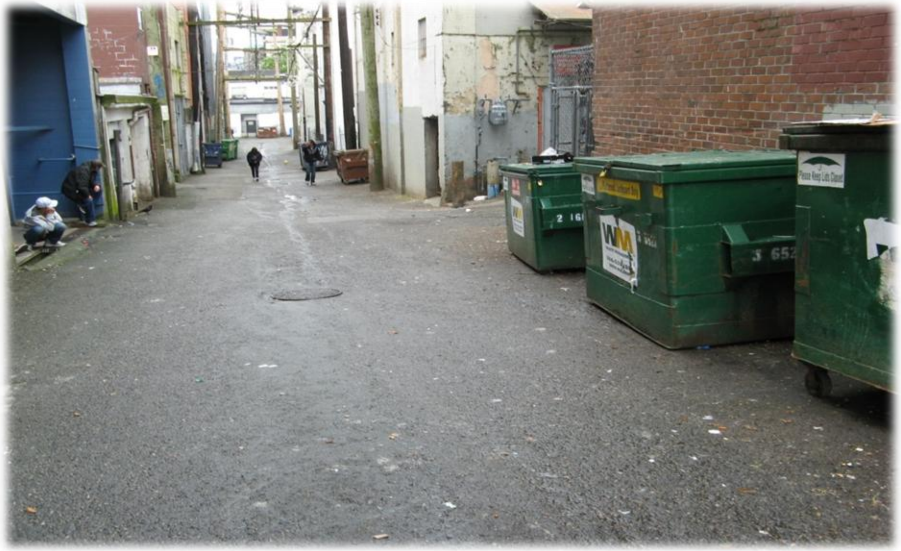

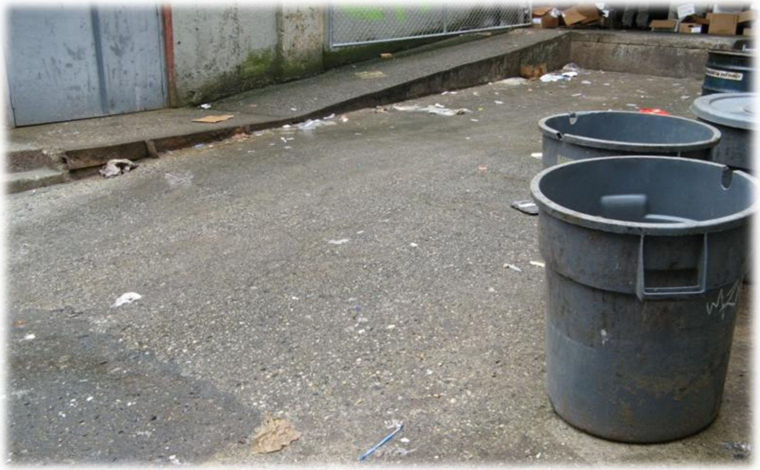

Grounds Condition

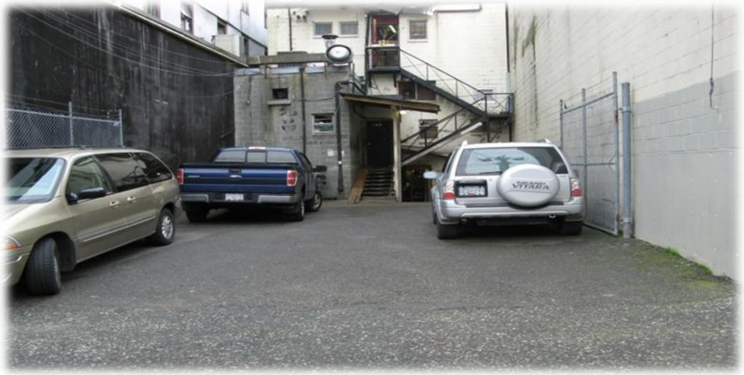

<u>Good:</u> Looks clean and well kept.

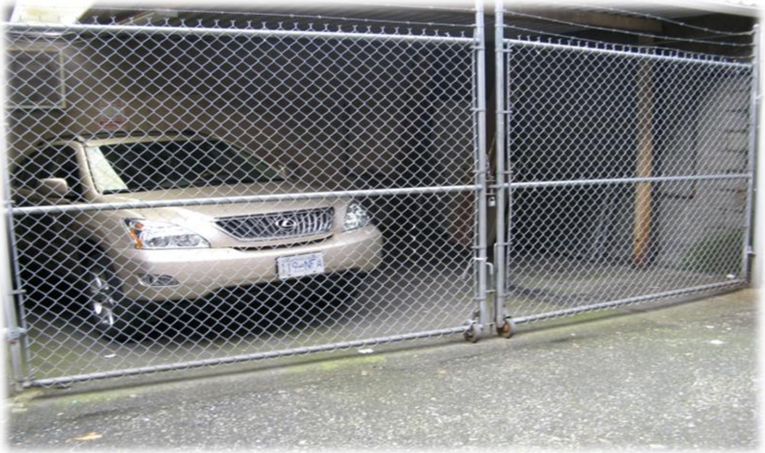

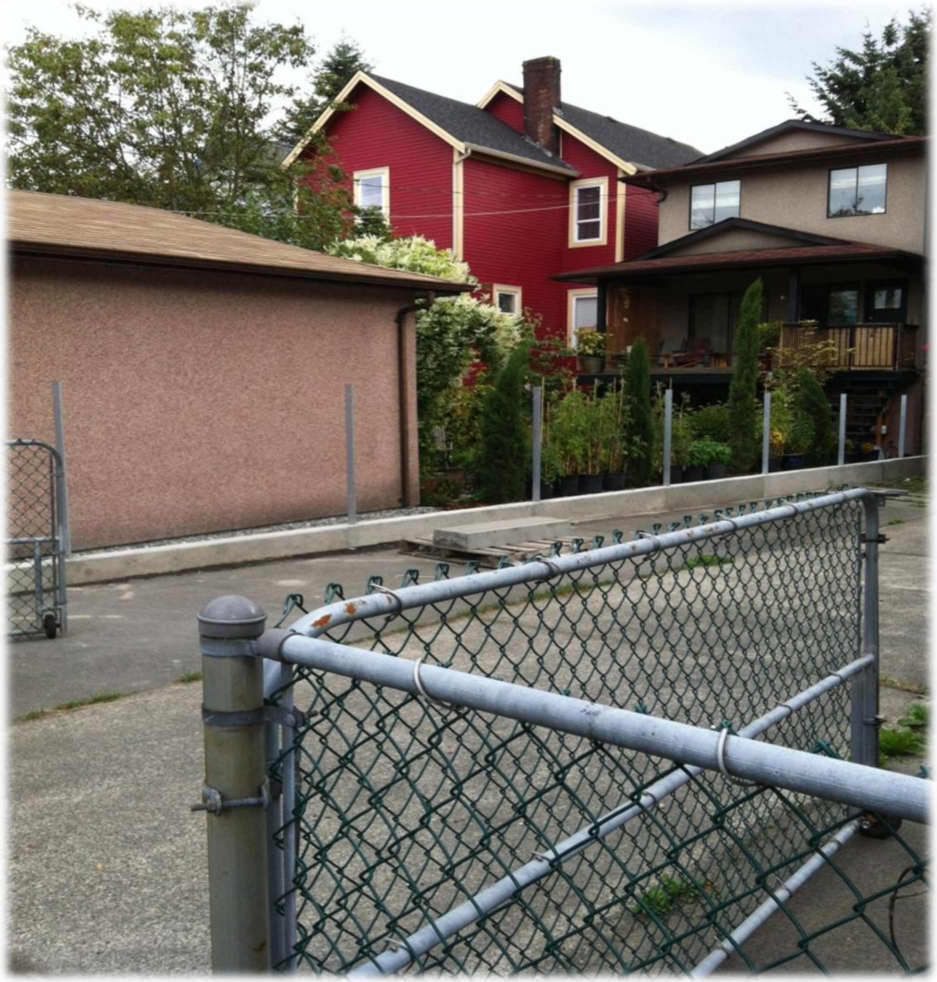

Grounds Condition

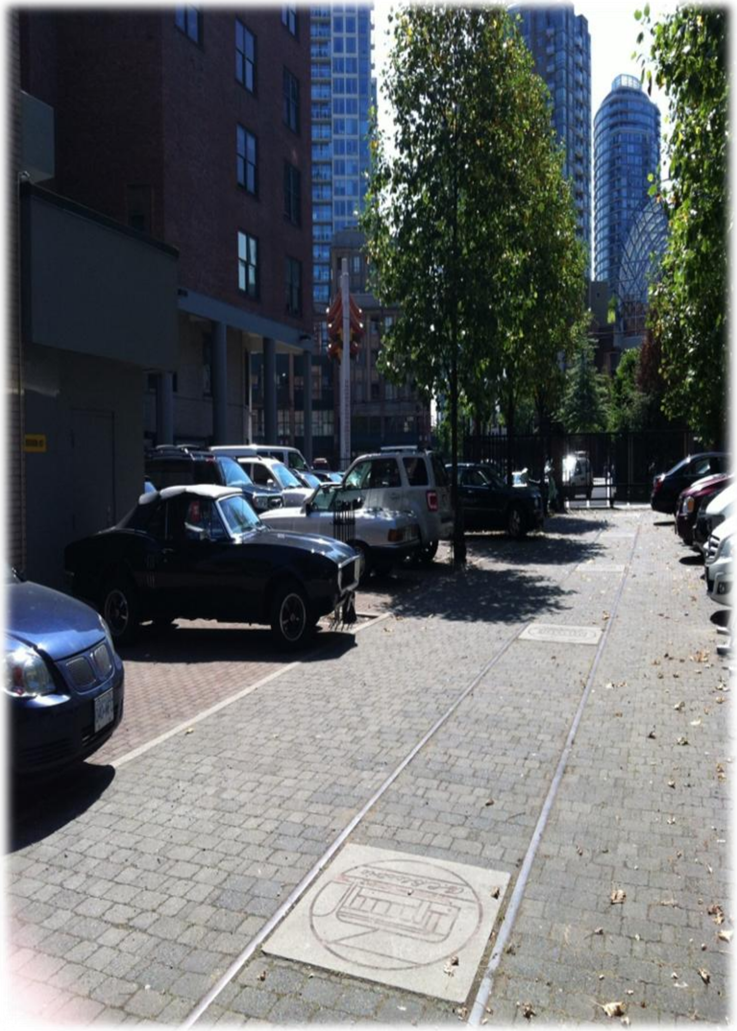

Excellent: Immaculate/near perfect condition. Shows that extra care and effort has been directed towards the upkeep.

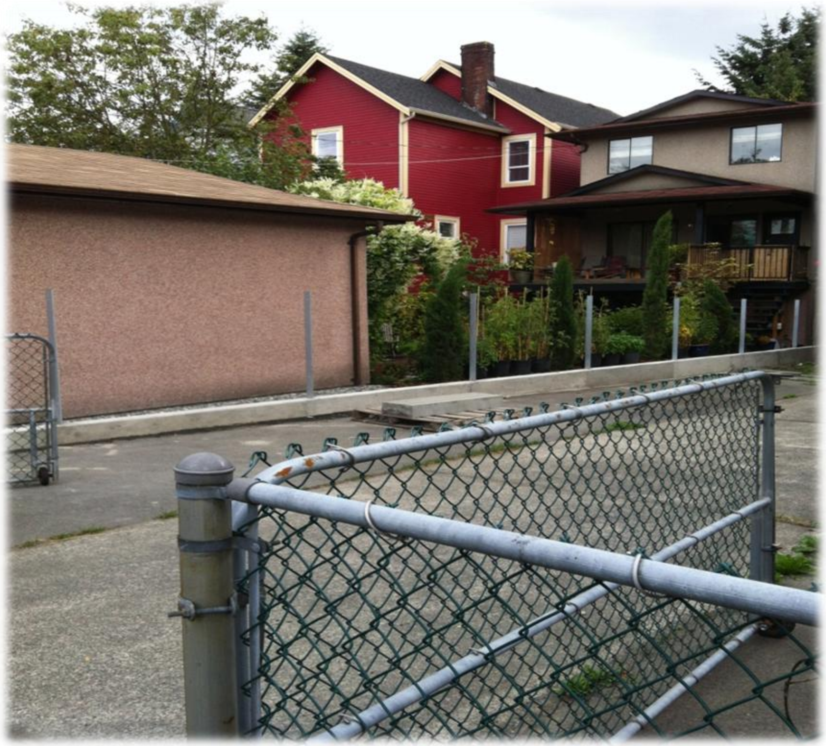

Green Space

For an area to be considered a green space it must:

1) Show significant plant growth (i.e., areas with sparse grass or weeds should NOT be included) and

2) Occupy a significant proportion of the property parcel (i.e., very small areas of plant growth in an otherwise build up parcel are not included).

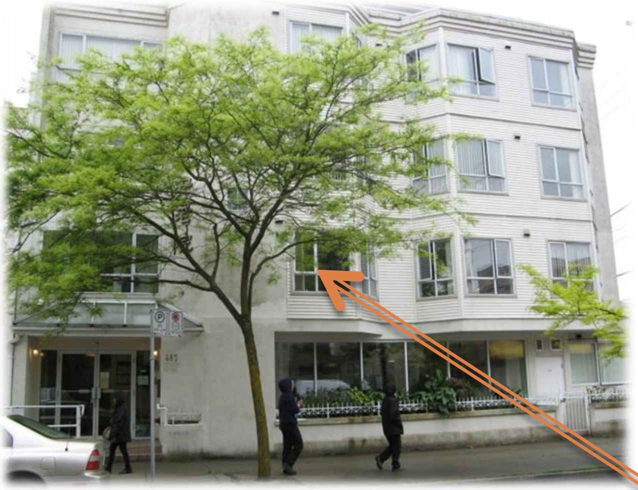

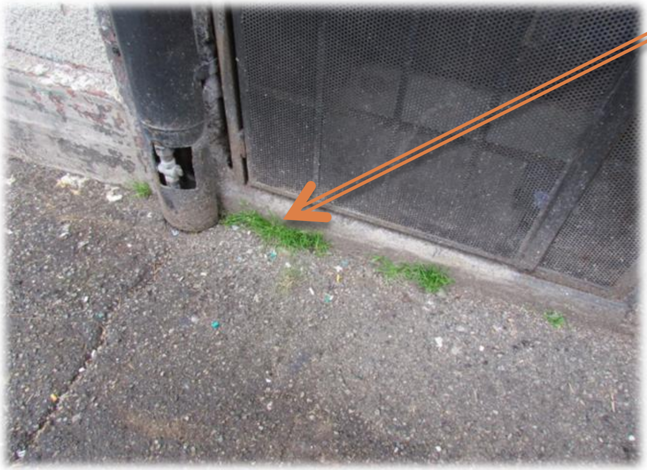

NOT Green space

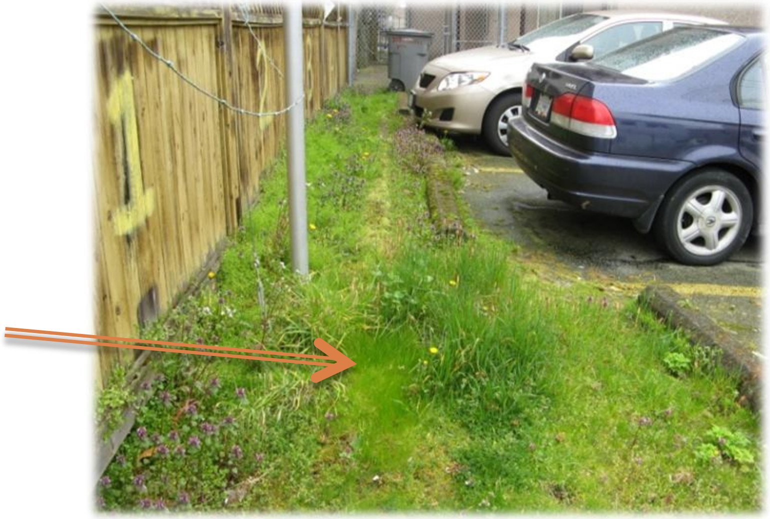

Green space

Green Space

<u>Unkempt green space:</u> an area that is overgrown by vegetation and/or does not appear to receive regular maintenance

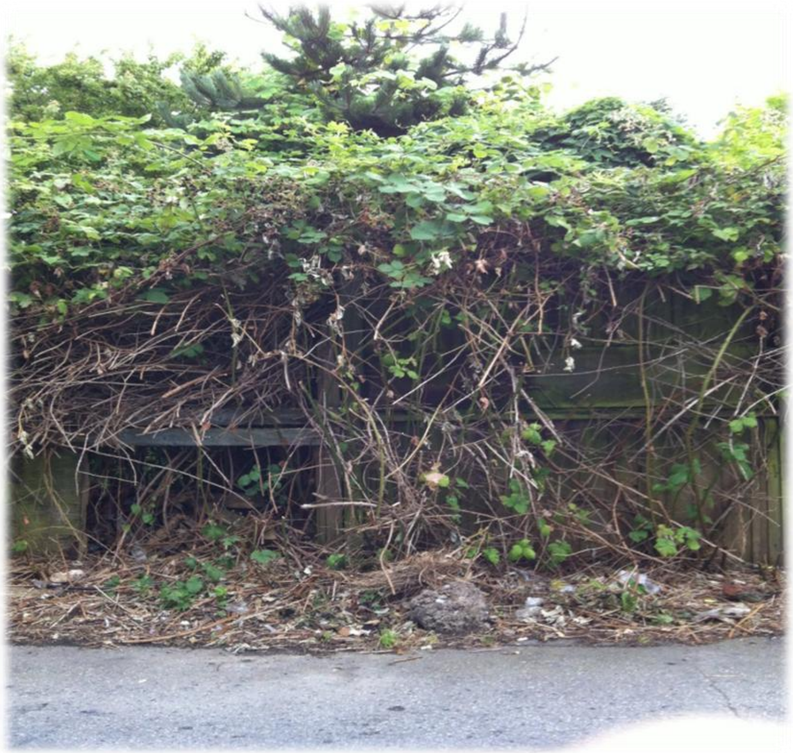

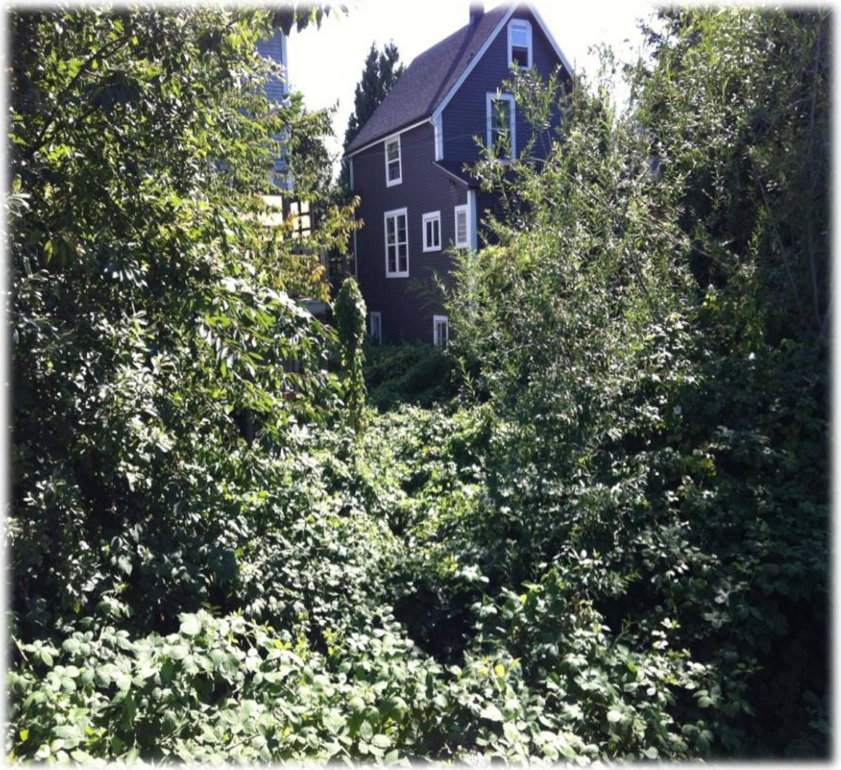

Green Space

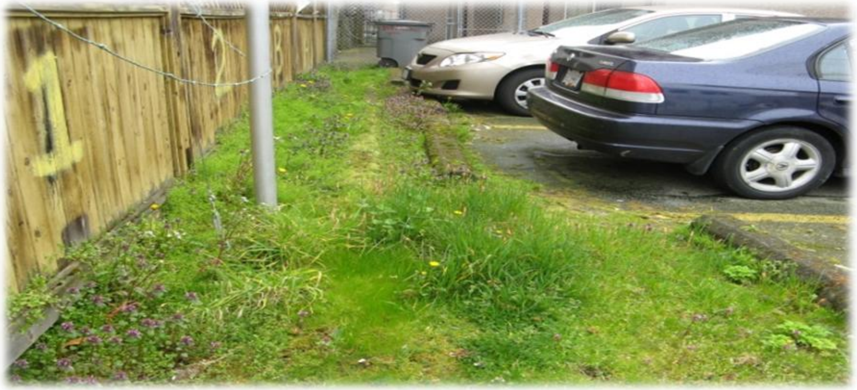

Well-kept green space: a green space that receives regular maintenance. Includes lawns, non-food gardens and or other landscaped areas.

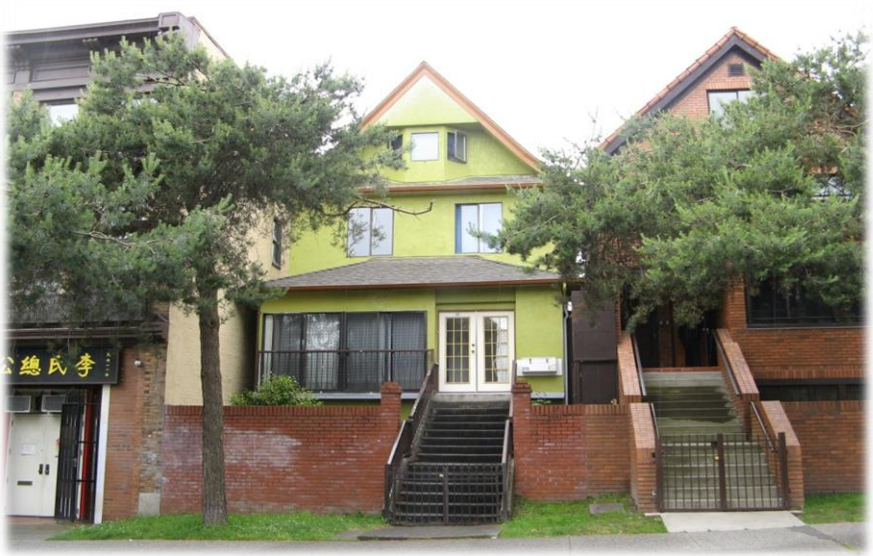

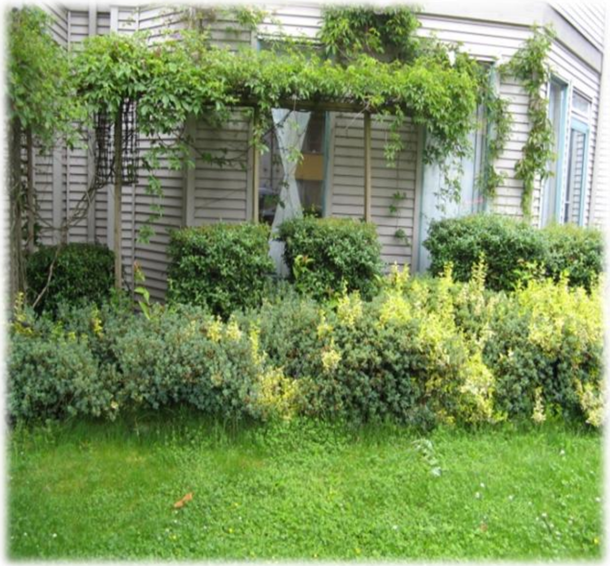

Green Space

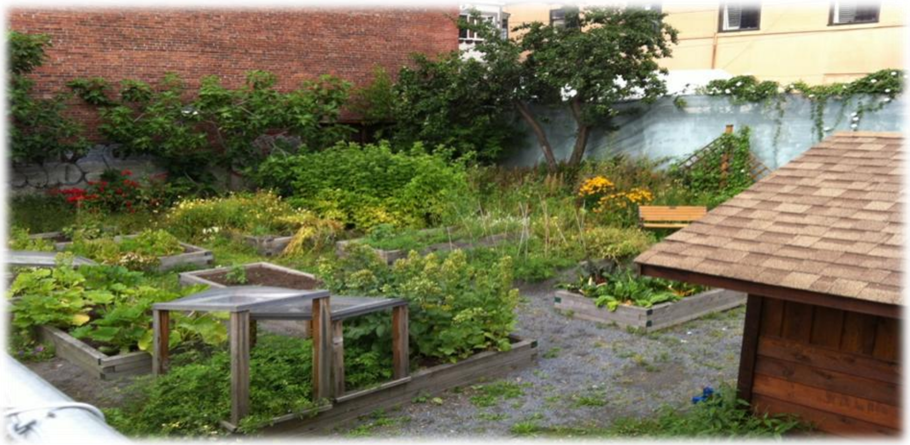

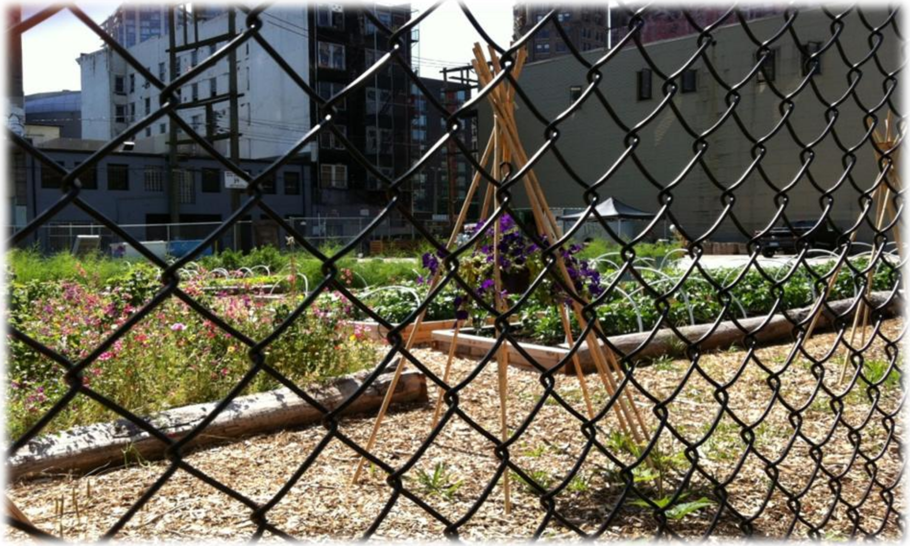

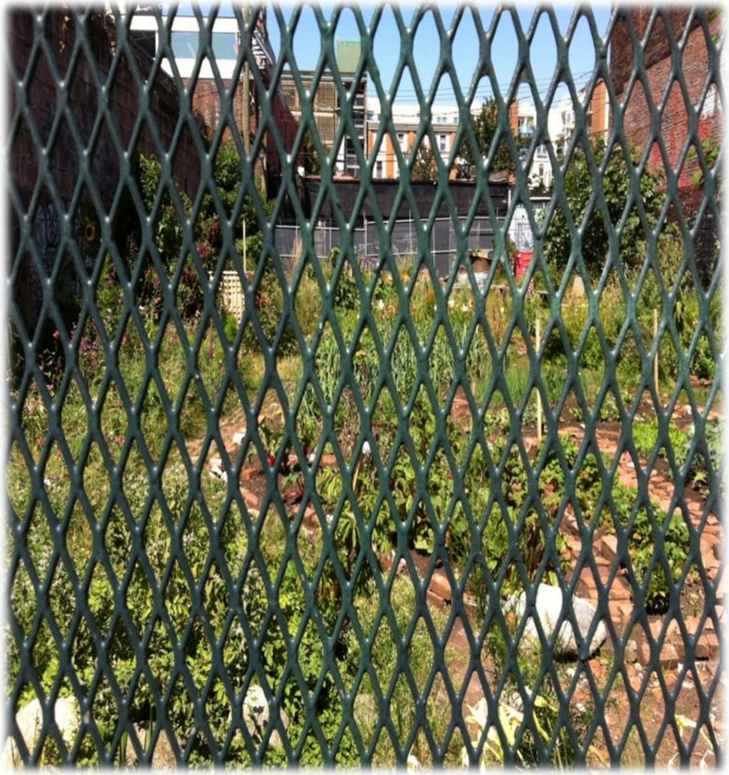

Food Garden: a green space that is in any way geared towards the production of produce.

Paved Surfaces

- Defined as any surface in a block that is paved
- Rated for its general state of repair/disrepair

Paved Surfaces

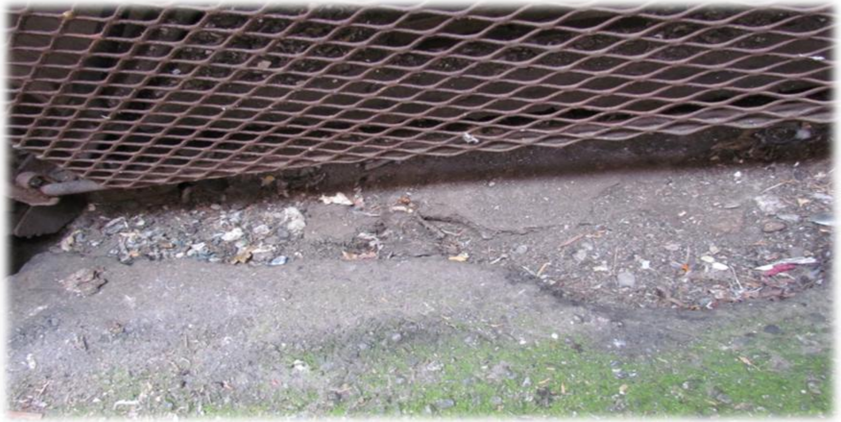

Poor: Frequent/severe deep cracking of pavement (cracking sufficient to permit rat burrowing). Alley surface is broken up and in need of serious repair.

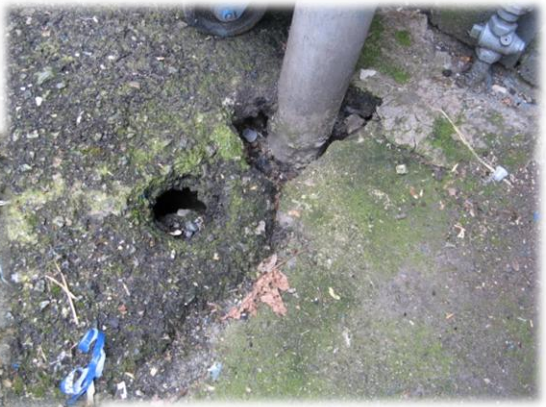

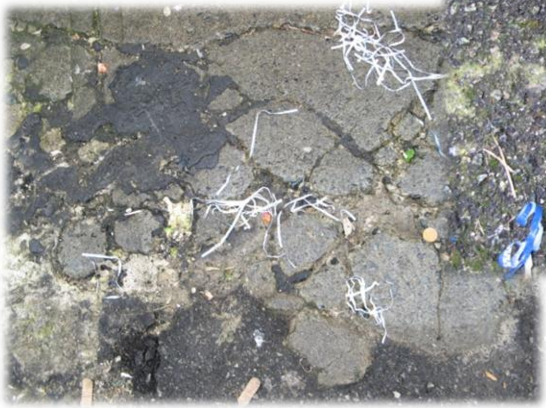

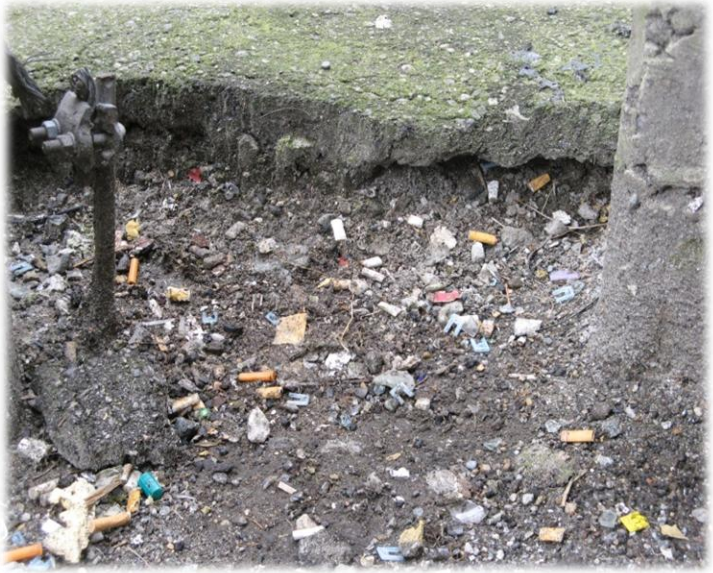

Paved Surfaces

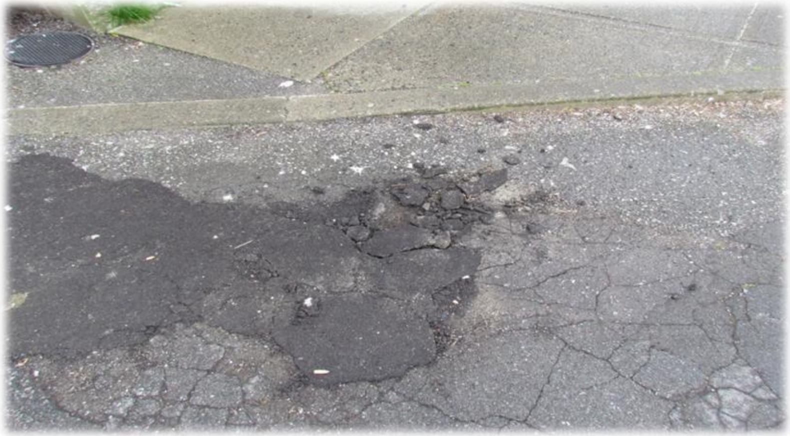

Fair: Moderate amount of cracked pavement. Cracking is mainly superficial (unlikely to promote rat harborage).

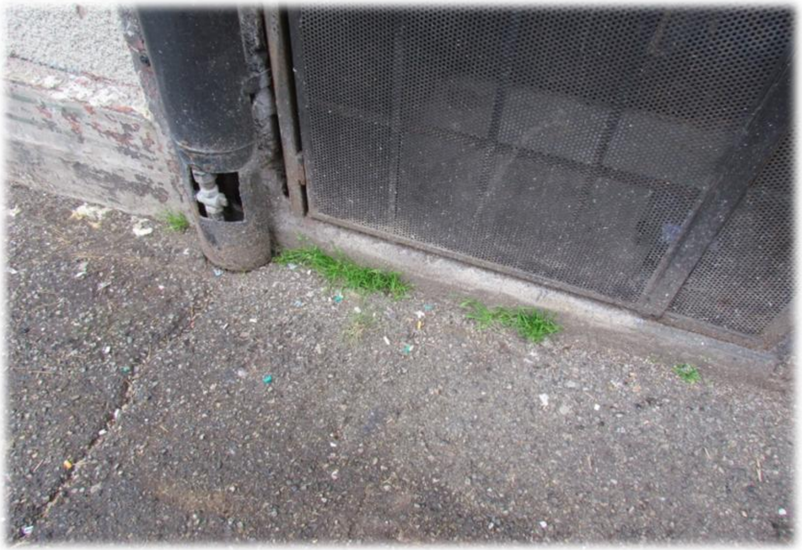

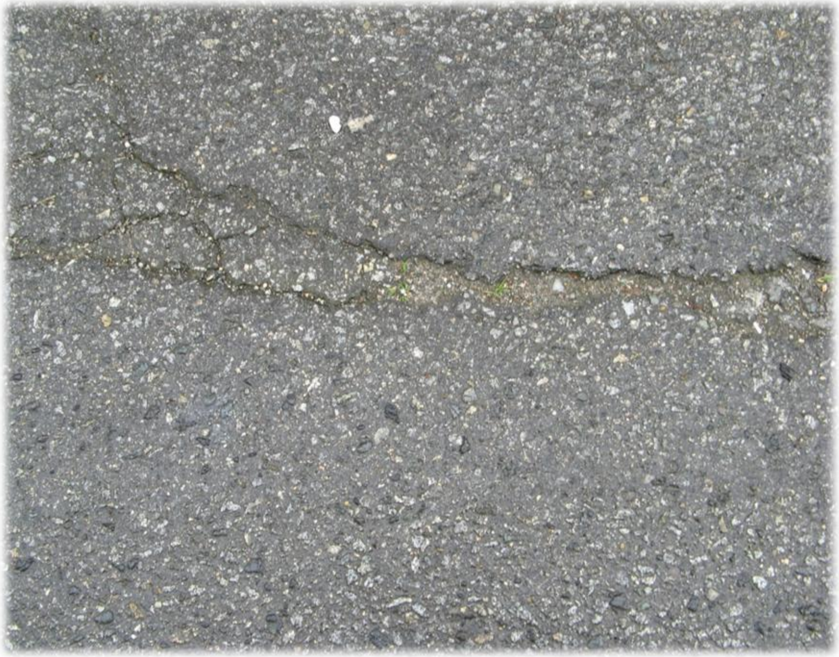

Paved Surfaces

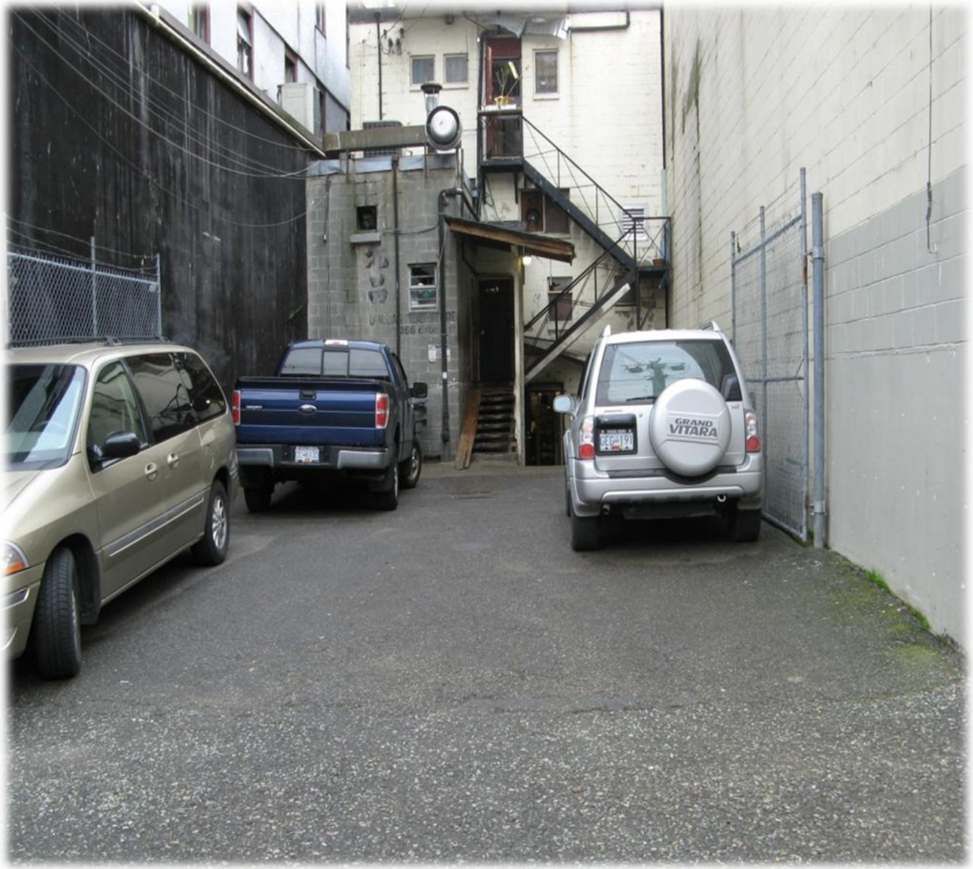

<u>Good</u>: Generally even surface with minimal cracking.

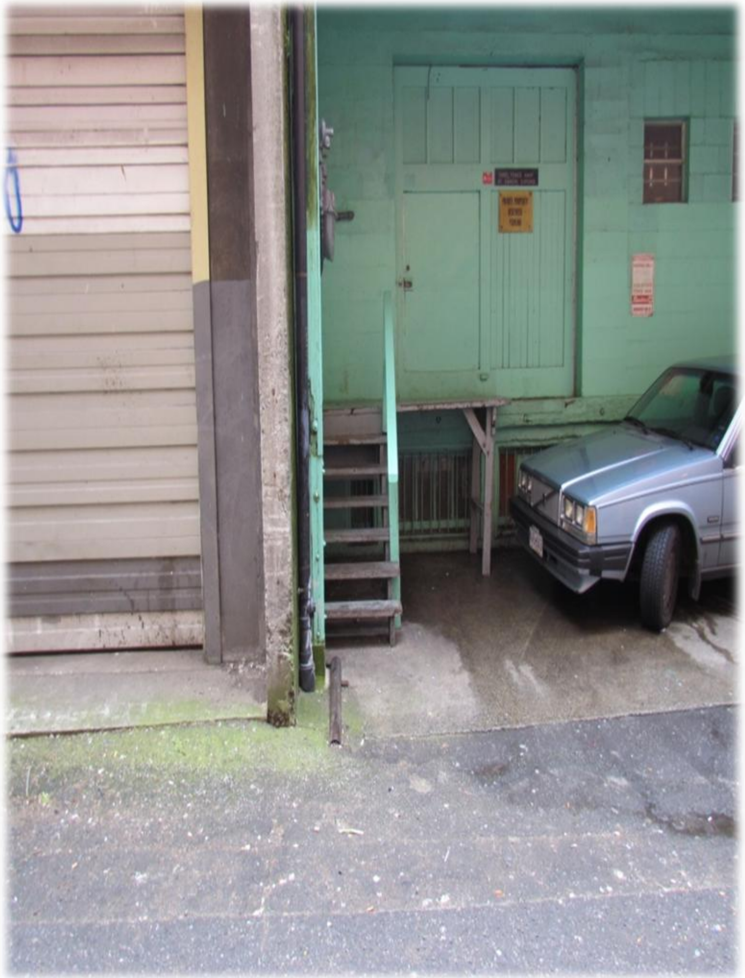

Non-Paved Surfaces

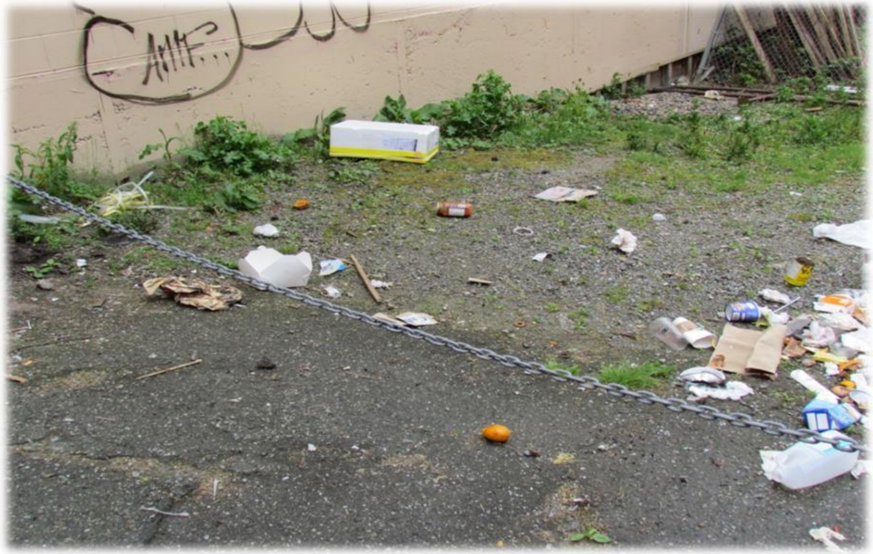

Defined as any space with 1m of the alley that is not paved.

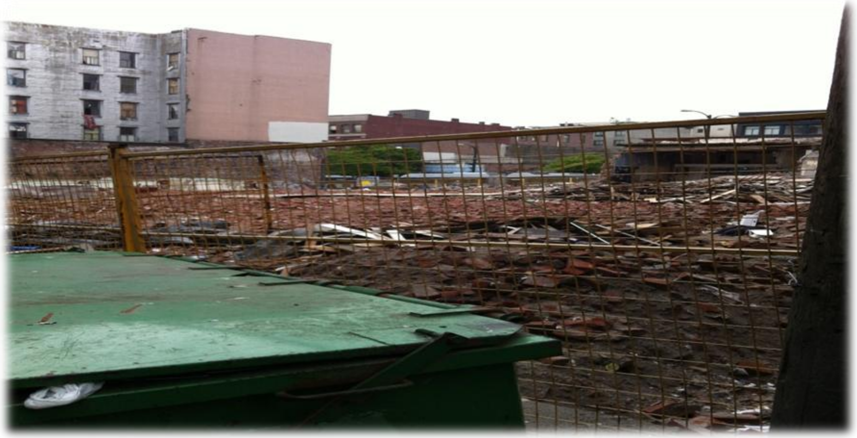

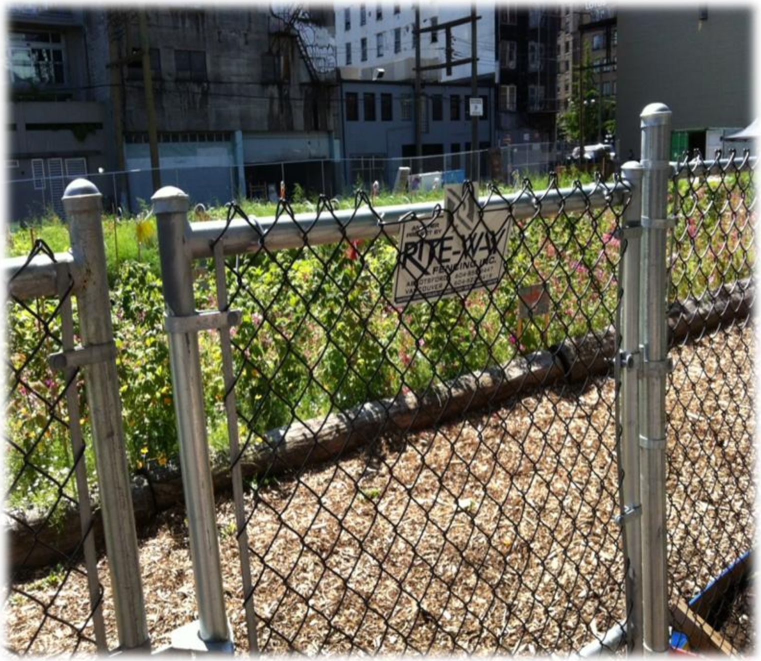

Presence of Garbage in/near Alley

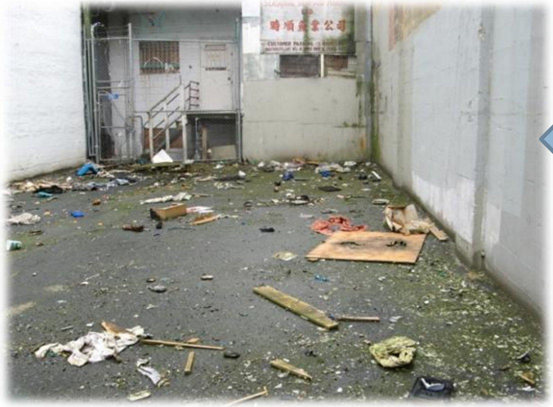

A lot of waste when there is a high volume of waste and/or waste is present throughout a large proportion of the alley.

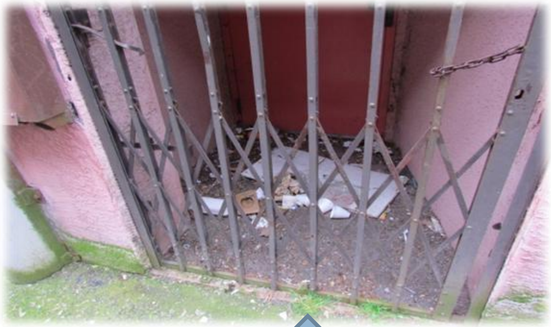

A little waste is when there is a very low volume of waste or the waste is confined to a small area.

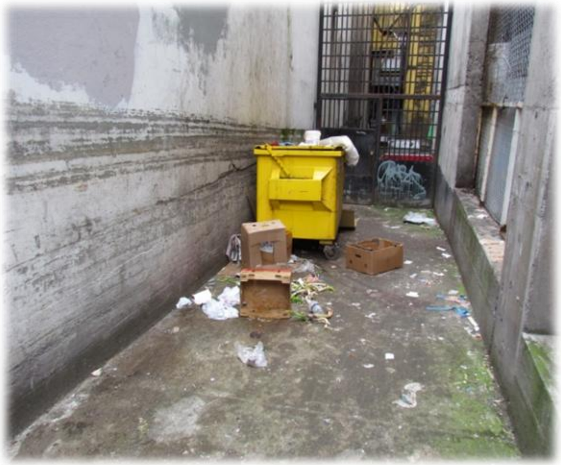

Some waste is somewhere between a little and a lot.

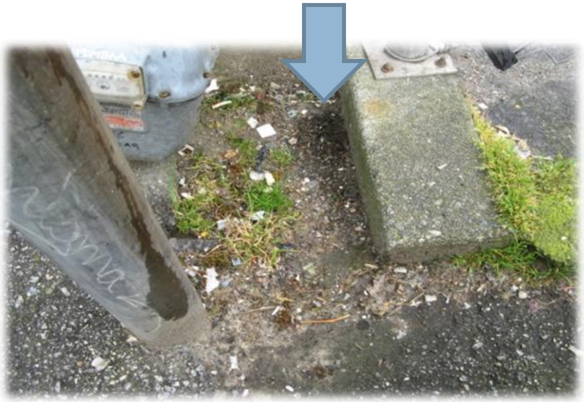

Overflowing Garbage Receptacles

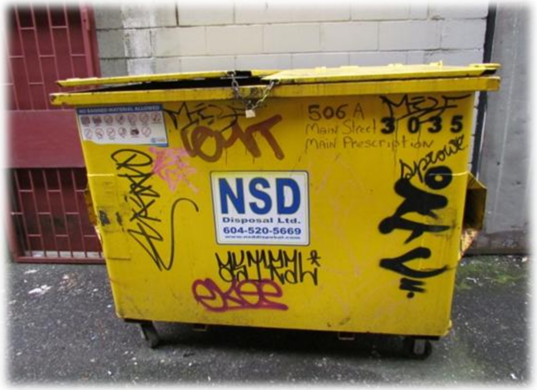

None

A little

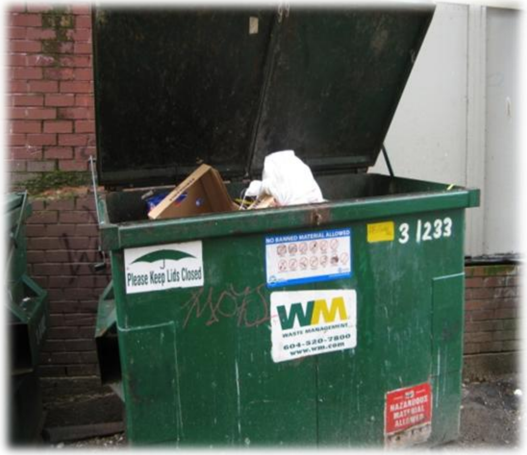

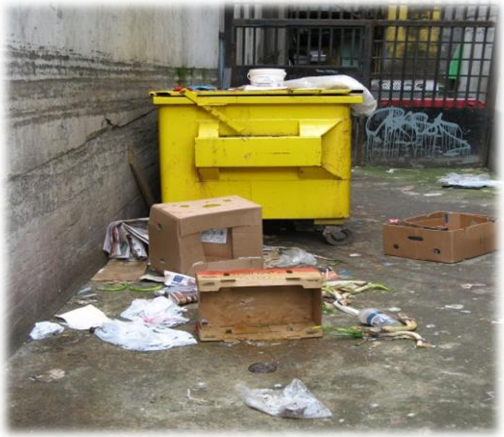

Some

A lot

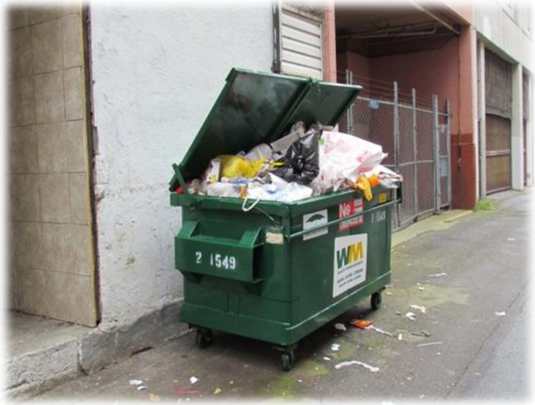

Garbage Receptacles

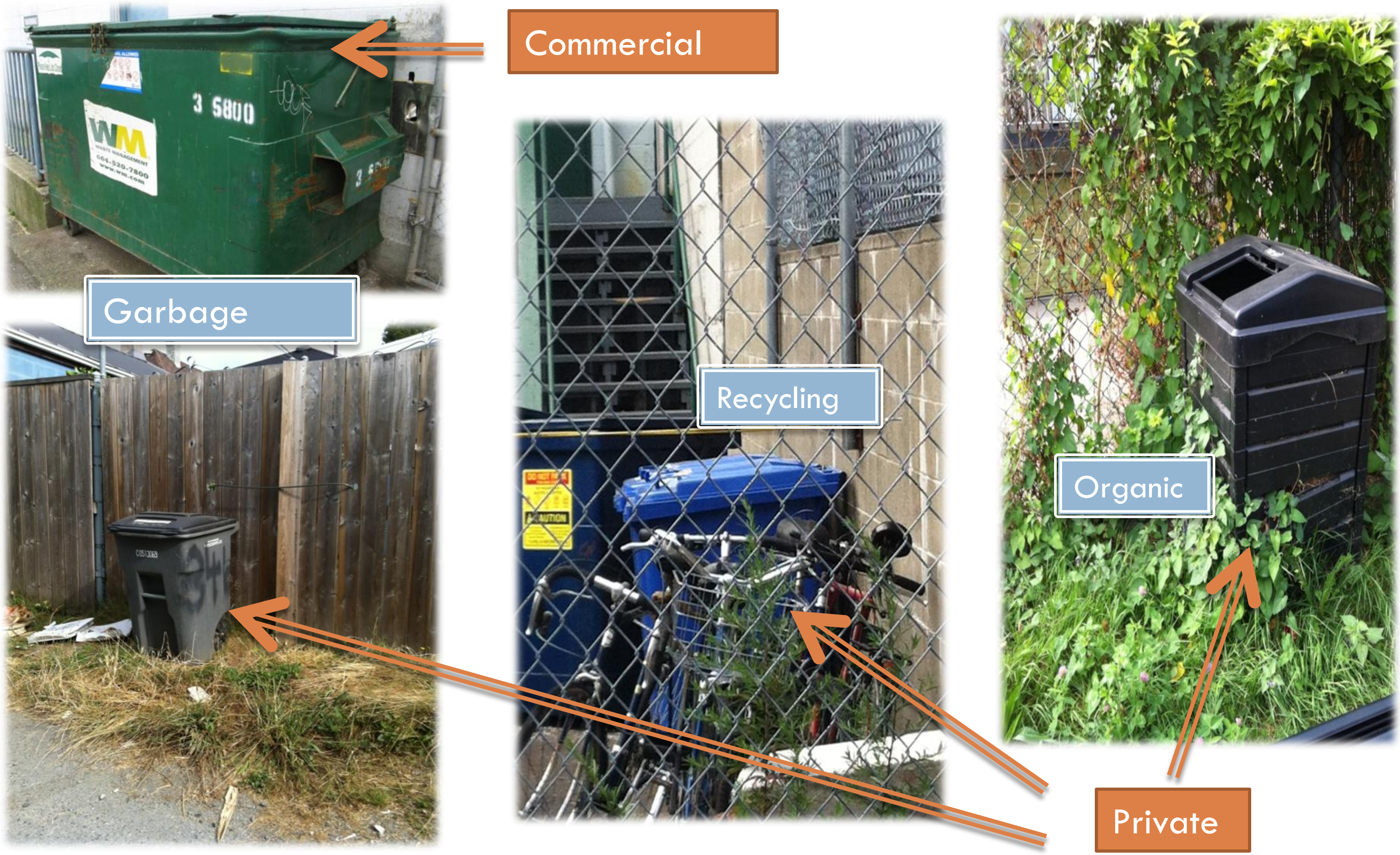

Rat Holes

Burrows in soil or pavement that look like they could have been made by a rat.

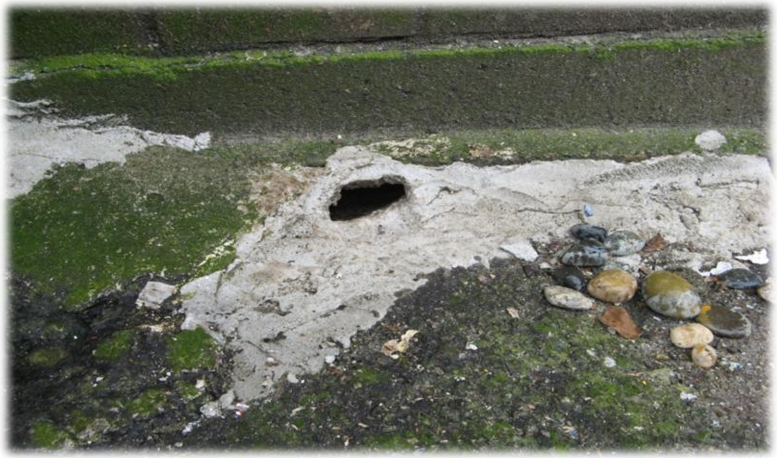

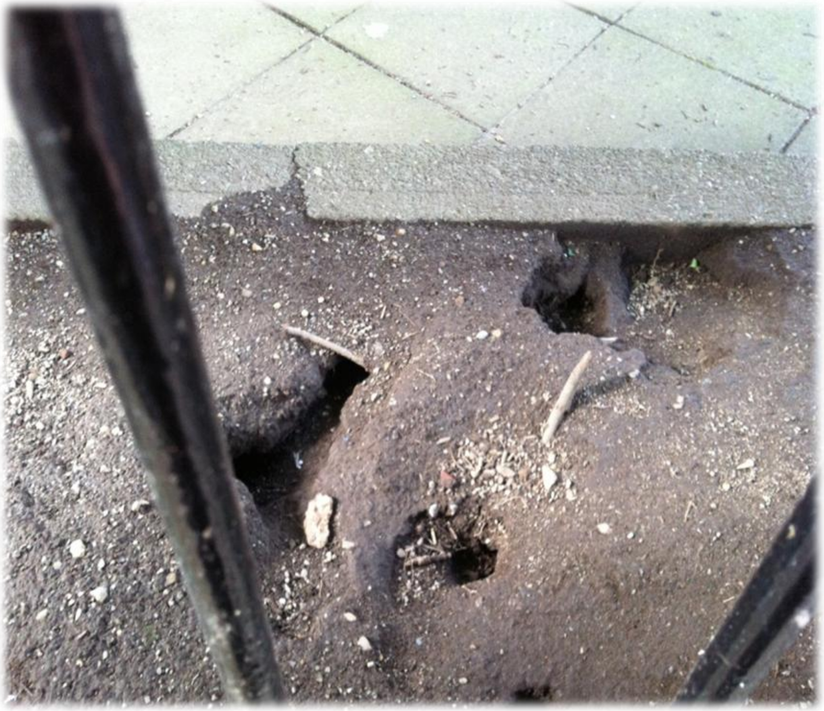

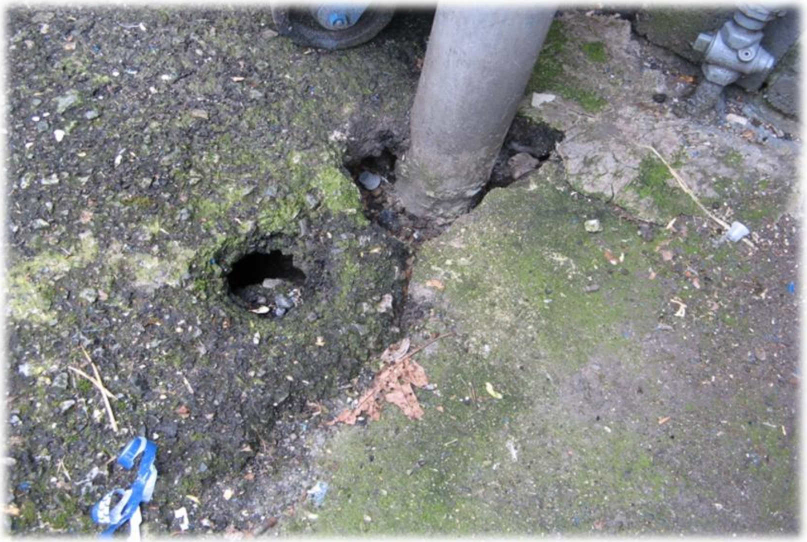

Rat Corridors

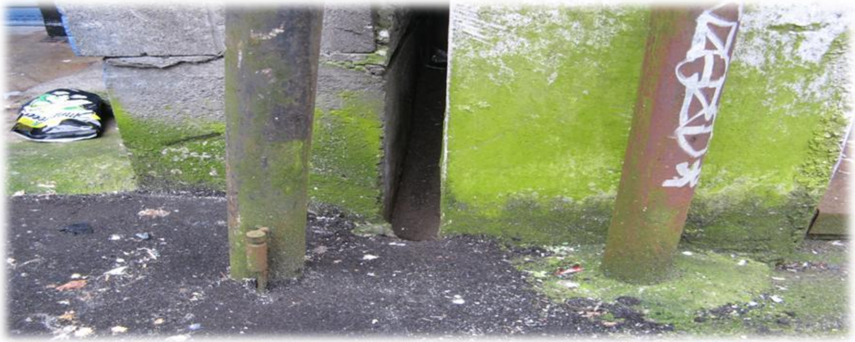

Defined as a narrow space in between buildings that is wide enough for rats to use as transportation routes.

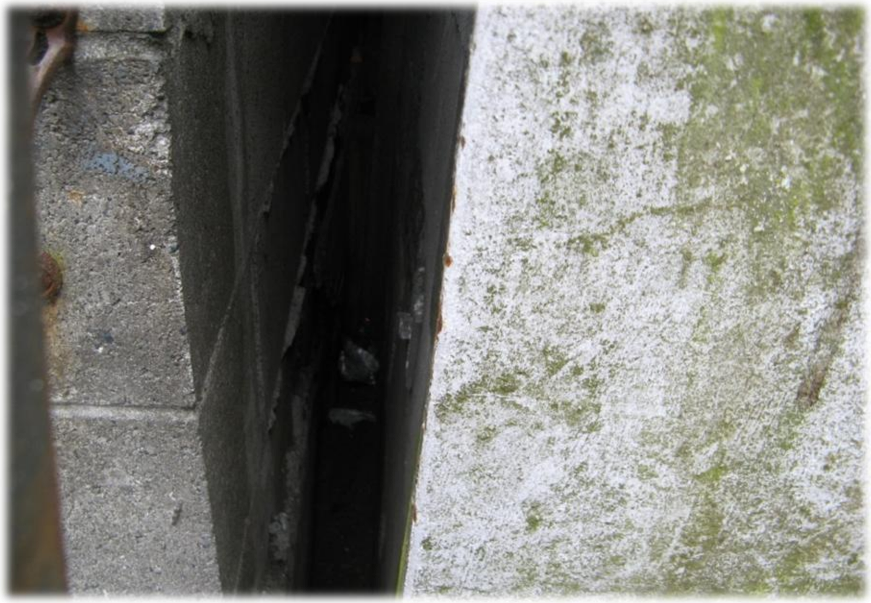

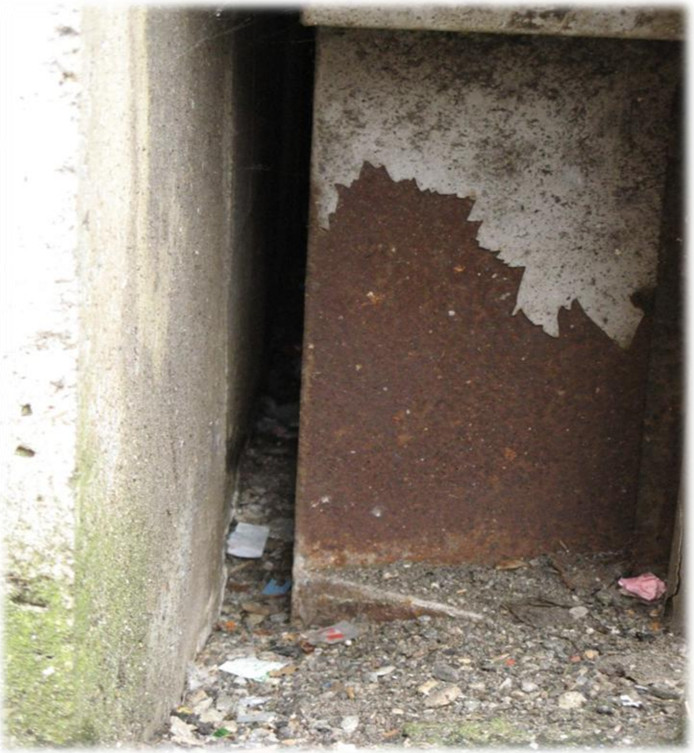

Supplement: Appendix S2 — Guidance document for the systematic environmental observation tool. (PDF) [file pone.0097776.s002.pdf]
