# Supplementary material for: A Mixed Methods Approach to Exploring the Relationship between Norway Rat (Rattus norvegicus) Abundance and Features of the Urban Environment in an Inner-City Neighborhood of Vancouver, Canada
Source: PLoS One. 2014 May 15;9(5):e97776. doi: 10.1371/journal.pone.0097776 (PMC4022650; doi:10.1371/journal.pone.0097776)
Supplement: Table S1 — Quantitative predictors generated from the environmental observation tool. (DOCX) [file pone.0097776.s003.docx]

Table S1: Quantitative predictors generated from the environmental observation tool.

| **Item/ Variable #** | | | **Variable description** |
| --- | --- | --- | --- |
| ***Land use variables*** | | | |
|  | 1 | **Predominant land use** on the block | |
| *^a^ | 2 | Proportion of block occupied by **residential** parcels | |
| * | 3 | Proportion of block occupied by **commercial** parcels | |
| * | 4 | Proportion of block occupied by **industrial** parcels | |
| * | 5 | Proportion of block occupied by **institutional** parcels | |
|  | 6 | Proportion of block occupied by **green space** | |
| * | 7 | Proportion of block occupied by **vacant** parcels | |
|  | 8 | Proportion of block occupied by parcels **under demolition** | |
| * | 9 | Proportion of block occupied by parcels **under construction** | |
| * | 10 | Proportion of block occupied by **abandoned** parcels | |
| * | 11 | Proportion of block occupied by **open** parcels | |
|  | 12 | Proportion of block occupied by **other land uses** | |
| ***Residential density variables*** | | | |
|  | 13 | **Predominant housing type** on the block | |
| * | 14 | Proportion of block occupied by **single family houses** | |
|  | 15 | Proportion of block occupied by **duplexes/rowhouses** | |
| * | 16 | Proportion of block occupied by **low-rise apartments** | |
| * | 17 | Proportion of block occupied by **mid-rise apartments** | |
|  | 18 | Proportion of block occupied by **high-rise apartments** | |
| * | 19 | Proportion of block occupied by **housing over commercial** | |
| * | 20 | Proportion of block occupied by **buildings not associated with food** | |
| * | 21 | Proportion of block occupied by **restaurants** | |
| * | 22 | Proportion of block occupied by **groceries** | |
| * | 23 | Proportion of block occupied by **industrial food establishments** | |
| * | 24 | Proportion of block occupied by **other food establishments** | |
| ***Property condition variables*** | | | |
|  | 25 | **General building condition** on the block | |
| * | 26^b^ | Proportion of block occupied by **buildings in extremely poor condition** | |
| * | 27^b^ | Proportion of block occupied by **buildings in poor condition** | |
| * | 28^b^ | Proportion of block occupied by **buildings in fair condition** | |
| * | 29^b^ | Proportion of block occupied by **buildings in good condition** | |
| * | 30^b^ | Proportion of block occupied by **buildings in excellent condition** | |
|  | 31 | **General grounds condition** on the block | |
| * | 32^c^ | Proportion of block occupied by **grounds in extremely poor condition** | |
| * | 33^c^ | Proportion of block occupied by **grounds in poor condition** | |
| * | 34^c^ | Proportion of block occupied by **grounds in fair condition** | |
| * | 35^c^ | Proportion of block occupied by **grounds in good condition** | |
| * | 36^c^ | Proportion of block occupied by **grounds in excellent condition** | |
| ***Green space variables*** | | | |
| * | 37 | Proportion of block occupied by **green space** | |
| * | 38 | Proportion of block occupied by **unkempt green space** | |
| * | 39 | Proportion of block occupied by **well kept green space** | |
| * | 40 | Proportion of block occupied by **food gardens** | |
| ***Alley surface variables*** | | | |
|  | 41 | **General condition of paved surfaces** in the alley | |
| * | 42^d^ | Proportion of **alley surface in poor condition** | |
| * | 43^d^ | Proportion of **alley surface in fair condition** | |
| * | 44^d^ | Proportion of **alley surface in good condition** | |
| * | 45 | Proportion of alley bordered by **non-paved surface** | |
| * | 46 | Number of **rat holes** | |
| * | 47 | Number of **rat corridors** | |
| ***Waste variables*** | | | |
| * | 48 | Amount of **garbage/trash/junk/litter** | |
| * | 49 | Amount of **overflowing garbage receptacles** | |
| * | 50 | Number of **commercial garbage receptacles** | |
| * | 51 | Number of **private garbage receptacles** | |
| * | 52 | Number of **commercial recycling receptacles** | |
| * | 53 | Number of **private recycling receptacles** | |
|  | 54 | Number of **commercial organic receptacles** | |
| * | 55 | Number of **private organic receptacles** | |
| * | 56 | Presence of **strong odors** | |
| ***Alley usage variables*** | | | |
| * | 57 | Amount of **loitering** | |
| * | 58 | Amount of **transport** | |

^a^Asterisk indicates variables for considered for inclusion in statistical modeling.

^b^Combined to create variable 26t30.

^c^Combined to create variable 32t36.

^d^Combined to create variable 42t44.
