# Supplementary material for: A Mixed Methods Approach to Exploring the Relationship between Norway Rat (Rattus norvegicus) Abundance and Features of the Urban Environment in an Inner-City Neighborhood of Vancouver, Canada
Source: PLoS One. 2014 May 15;9(5):e97776. doi: 10.1371/journal.pone.0097776 (PMC4022650; doi:10.1371/journal.pone.0097776)
Supplement: Table S2 — Bivariate relationships between features of the urban environment and relative abundance of Norway rats ( Rattus norvegicus ). Zero-inflated negative binomial model for the number of rats trapped in a city block offset by the natural logarithm of the trap effort in that block. (DOCX) [file pone.0097776.s004.docx]

Table S2: Bivariate relationships between features of the urban environment and relative abundance of Norway rats (*Rattus norvegicus*). Zero-inflated negative binomial model for the number of rats trapped in a city block offset by the natural logarithm of the trap effort in that block.

| **Variable #** | **Variable description** | **β** | **SE** | **P-Value** |  |
| --- | --- | --- | --- | --- | --- |
| ***Count Model (negative binomial with log link)***^a^ | |  |  |  |  |
| 2 | Proportion of block occupied by **residential** parcels | -0.01 | 0.28 | 0.96 |  |
| 3 | Proportion of block occupied by **commercial** parcels | 0.44 | 0.25 | 0.07 |  |
| 4 | Proportion of block occupied by **industrial** parcels | -0.74 | 0.30 | 0.01 | * |
| 5 | Proportion of block occupied by **institutional** parcels | 1.05 | 0.87 | 0.23 |  |
| 7 | Proportion of block occupied by **vacant** parcels | 0.08 | 0.48 | 0.09 |  |
| 9 | Proportion of block occupied by parcels **under construction** | 0.10 | 0.66 | 0.87 |  |
| 10 | Proportion of block occupied by **abandoned** parcels | 0.09 | 0.49 | 0.84 |  |
| 11 | Proportion of block occupied by **open** parcels | -0.22 | 0.49 | 0.65 |  |
| 14 | Proportion of block occupied by **single family houses** | -1.11 | 0.25 | <0.01 | * |
| 16 | Proportion of block occupied by **low-rise apartments** | -0.14 | 0.41 | 0.74 |  |
| 17 | Proportion of block occupied by **mid-rise apartments** | -0.15 | 0.46 | 0.75 |  |
| 19 | Proportion of block occupied by **housing over commercial** | 0.81 | 0.28 | <0.01 | * |
| 20 | Proportion of block occupied by **buildings not associated with food** | -0.23 | 0.43 | 0.58 |  |
| 21 | Proportion of block occupied by **restaurants** | 0.61 | 0.51 | 0.23 |  |
| 22 | Proportion of block occupied by **groceries** | 0.82 | 0.33 | 0.01 | * |
| 23 | Proportion of block occupied by **industrial food establishments** | -0.74 | 0.42 | 0.08 |  |
| 24 | Proportion of block occupied by **other food establishments** | 0.36 | 0.53 | 0.50 |  |
| 26t30 | General **building condition** | -2.27 | 0.99 | 0.02 | * |
| 32t36 | General **grounds condition** | -1.74 | 0.50 | <0.01 | * |
| 37 | Proportion of block occupied by **green space** | -1.60 | 0.50 | <0.01 | * |
| 38 | Proportion of block occupied by **unkempt green space** | -0.83 | 0.50 | 0.10 |  |
| 39 | Proportion of block occupied by **well kept green space** | -1.35 | 0.44 | <0.01 | * |
| 40 | Proportion of block occupied by **food gardens** | -0.69 | 0.54 | 0.20 |  |
| 42t44 | General **alley surface condition** | -1.30 | 0.68 | 0.06 |  |
| 45 | Proportion of alley bordered by **non-paved surface** | -0.68 | 0.34 | 0.04 | * |
| 47 | Number of **rat corridors** | -0.22 | 0.20 | 0.25 |  |
| 48 | Amount of **garbage/trash/junk/litter** | 0.98 | 0.31 | <0.01 | * |
| 49 | Amount of **overflowing garbage receptacles** | 0.66 | 0.28 | 0.02 | * |
| 50 | Number of **commercial garbage receptacles** | 0.17 | 0.04 | <0.01 | * |
| 51 | Number of **private garbage receptacles** | -1.18 | 0.05 | <0.01 | * |
| 52 | Number of **commercial recycling receptacles** | 0.27 | 0.12 | 0.03 | * |
| 53 | Number of **private recycling receptacles** | 0.06 | 0.12 | 0.64 |  |
| 55 | Number of **private organic receptacles** | -1.16 | 0.49 | 0.02 | * |
| 56 | Presence of **strong odors** | 1.11 | 0.46 | 0.02 | * |
| 57 | Amount of **loitering** | 0.60 | 0.26 | 0.02 | * |
| 58 | Amount of **transport** | 0.20 | 0.44 | 0.65 |  |
| Fall vs. Summer | **Season** of trapping | 0.66 | 0.75 | 0.38 |  |
| Spring vs. Summer | **Season** of trapping | 0.51 | 0.68 | 0.46 |  |
| Winter vs. Summer | **Season** of trapping | 0.22 | 0.73 | 0.76 |  |
| ***Zero-Inflation Model (binomial with logit link)***^a^ | |  |  |  |  |
| 2 | Proportion of block occupied by **residential** parcels | -10.18 | 51.64 | 0.84 |  |
| 3 | Proportion of block occupied by **commercial** parcels | -10.41 | 57.01 | 0.86 |  |
| 4 | Proportion of block occupied by **industrial** parcels | 7.70 | 55.88 | 0.89 |  |
| 5 | Proportion of block occupied by **institutional** parcels | -4.04 | 1.97 | 0.04 | * |
| 7 | Proportion of block occupied by **vacant** parcels | -1.26 | 6.37 | 0.84 |  |
| 9 | Proportion of block occupied by parcels **under construction** | -7.00 | 85.28 | 0.94 |  |
| 10 | Proportion of block occupied by **abandoned** parcels | -2.21 | 8.10 | 0.79 |  |
| 11 | Proportion of block occupied by **open** parcels | 1.10 | 4.56 | 0.81 |  |
| 14 | Proportion of block occupied by **single family houses** | 6.71 | 39.97 | 0.87 |  |
| 16 | Proportion of block occupied by **low-rise apartments** | -8.63 | 85.95 | 0.92 |  |
| 17 | Proportion of block occupied by **mid-rise apartments** | -2.51 | 7.81 | 0.75 |  |
| 19 | Proportion of block occupied by **housing over commercial** | -1.93 | 1.02 | 0.06 |  |
| 20 | Proportion of block occupied by **buildings not associated with food** | 3.65 | 6.00 | 0.54 |  |
| 21 | Proportion of block occupied by **restaurants** | -2.42 | 5.49 | 0.66 |  |
| 22 | Proportion of block occupied by **groceries** | -9.75 | 78.41 | 0.90 |  |
| 23 | Proportion of block occupied by **industrial food establishments** | 13.30 | 41.89 | 0.75 |  |
| 24 | Proportion of block occupied by **other food establishments** | -8.02 | 83.650.92 | 0.92 |  |
| 26t30 | General **building condition** | 25.13 | 17.40 | 0.15 |  |
| 32t36 | General **grounds condition** | 66.96 | 142.33 | 0.64 |  |
| 37 | Proportion of block occupied by **green space** | 9.62 | 56.86 | 0.87 |  |
| 38 | Proportion of block occupied by **unkempt green space** | -1.28 | 4.08 | 0.75 |  |
| 39 | Proportion of block occupied by **well kept green space** | 7.97 | 43.20 | 0.85 |  |
| 40 | Proportion of block occupied by **food gardens** | 3.94 | 28.80 | 0.89 |  |
| 42t44 | General **alley surface condition** | 23.31 | 41.76 | 0.58 |  |
| 45 | Proportion of alley bordered by **non-paved surface** | 13.17 | 34.01 | 0.70 |  |
| 47 | Number of **rat corridors** | -1.15 | 1.69 | 0.50 |  |
| 48 | Amount of **garbage/trash/junk/litter** | -1.63 | 2.10 | 0.43 |  |
| 49 | Amount of **overflowing garbage receptacles** | -0.31 | 1.09 | 0.78 |  |
| 50 | Number of **commercial garbage receptacles** | -0.82 | 0.87 | 0.35 |  |
| 51 | Number of **private garbage receptacles** | 0.34 | 0.39 | 0.38 |  |
| 52 | Number of **commercial recycling receptacles** | 2.53 | 10.80 | 0.82 |  |
| 53 | Number of **private recycling receptacles** | -9.42 | 84.72 | 0.91 |  |
| 55 | Number of **private organic receptacles** | 7.27 | 69.60 | 0.92 |  |
| 56 | Presence of **strong odors** | 6.43 | 52.80 | 0.90 |  |
| 57 | Amount of **loitering** | -2.61 | 1.26 | 0.04 | * |
| 58 | Amount of **transport** | -7.44 | 69.26 | 0.92 |  |
| Fall vs. Summer | **Season** of trapping | -19.41 | 3897.00 | 0.99 |  |
| Spring vs. Summer | **Season** of trapping | -11.82 | 111.79 | 0.92 |  |
| Winter vs. Summer | **Season** of trapping | -1.92 | 1.18 | 0.10 |  |

^a^Predictors included in the binary and count portions of the model independently.

^b^Statistically significant relationships (α = 0.05).
